# Postoperative Nodal Efficiency of the Lesional‐Hemispheric Hand Motor Area Increasing Potentially Facilitated Motor Recovery for SMA Syndrome

**DOI:** 10.1111/cns.70112

**Published:** 2024-11-12

**Authors:** Shimeng Weng, Zhaoting Meng, Lianwang Li, Jinshi Li, Xing Fan, Zhong Zhang, Yinyan Wang, Tao Jiang, Shengyu Fang

**Affiliations:** ^1^ Beijing Neurosurgical Institute Capital Medical University Beijing China; ^2^ Department of Neurosurgery Beijing Tiantan Hospital, Capital Medical University Beijing China; ^3^ Universal Medical Imaging Diagnostic Center Beijing China; ^4^ Tianjin Huanhu Hospital Tianjin China; ^5^ Department of Child and Adolescent Health, School of Public Health China Medical University Shenyang China; ^6^ Research Unit of Accurate Diagnosis, Treatment, and Translational Medicine of Brain Tumors Chinese Academy of Medical Sciences Beijing China; ^7^ Changping Laboratory Beijing China

**Keywords:** causal mediation, glioma, recovery time, supplementary motor area syndrome, transcranial magnetic stimulation

## Abstract

**Background:**

Supplementary motor area (SMA) syndrome commonly occurs after glioma resection and requires weeks to months of recovery.

**Methods:**

Thirty‐four glioma patients with SMA syndrome were reviewed and assigned to recovered and non‐recovered groups based on whether their motor function recovered on postoperative day 7. To validate the association between variations in nodal properties and recovery time, neuro‐navigated repetitive transcranial magnetic stimulation (nrTMS) was applied to stimulate potential nodes. Nine other patients (five nrTMS therapy and four sham‐nrTMS treatments) with SMA syndrome with unrecovered motor functions on postoperation day 7 were prospectively enrolled.

**Results:**

The potential nodes of the sensorimotor network related to recovery time were investigated using preoperative and postoperative resting‐state functional magnetic resonance imaging, graph theoretical analysis, and dynamic functional connectome analysis. Nodal efficiency of the lesional‐hemispheric upper limb region of BA 4 (A4ul_L) increased in the recovered group (preoperative, 0.472 ± 0.027; postoperative, 0.535 ± 0.020; *p* = 0.0006). The patients in the nrTMS therapy group quickly recovered (12.0 ± 1.6 days) compared to the sham‐nrTMS group (29.5 ± 3.8 days, *p* = 0.0024). Variations in A4ul_L nodal efficiency was negatively correlated with recovery time (*r* = −0.841; *p* = 0.0046).

**Conclusion:**

A4ul_L demonstrates enhanced postoperative nodal efficiency and shows therapeutic potential in SMA syndrome recovery, suggesting its viability as a therapeutic target.

## Graphical abstract

Hand‐motor‐related cortex on the lesional hemisphere (A4ul_L) increasing its nodal efficiency in postoperation was beneficial for SMA syndrome recovery. The A4ul_L may be a potential therapeutic target for SMA syndrome. Applying nrTMS with high‐frequency stimulation on the node A4ul_L will increase its nodal efficiency, but sham‐nrTMS won't. It may indicate that nrTMS stimulation may accelerate SMA syndrome recovery.

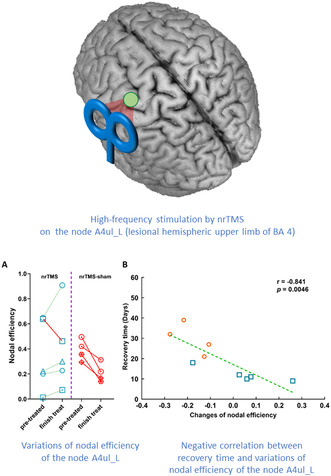

## 1 Introduction

Supplementary motor area (SMA) syndrome was first described by Laplane et al. in 1977 [1], which typically manifests within 24 h after glioma removal located in the SMA, presenting as transient hemiplegia [2, 3, 4]. Patients diagnosed with SMA syndrome often require weeks to months to regain motor functions [3]. There are observations to suggest that postoperative day 7 may be a good point in time to determine whether recovery has been delayed [2].

The understanding of the underlying mechanisms behind recovery from the SMA syndrome has historically been dominated by the hypothesis that the contralateral non‐lesioned SMA is gradually able to compensate for the function in the lesioned side through increased interhemispheric connectivity [5, 6]. More recently, emerging evidence suggest that there may be multiple routes to recovery, some of which may not be mediated by this mechanism [7, 8]. Resting‐state functional magnetic resonance imaging (rs‐fMRI) combined with graph theoretical analysis can precisely identify alterations in the structural organization of functional networks and elucidate their capacity for information transmission [9]. In our previous study, we found that the increased preoperative nodal efficiency and vulnerability of the lesional‐hemispheric hand‐motor area are risk factors for the occurrence of SMA syndrome [10]. However, the specific nodes and the nature of the topological property alterations within the sensorimotor network that influence SMA syndrome recovery remain unclear.

Neuro‐navigated repetitive transcranial magnetic stimulation (nrTMS) is a non‐invasive technique used to improve brain function rehabilitation by modulating the excitability of neural networks [11, 12]. Through neuroimaging, the causal relationship between changes in brain network excitability and corresponding clinical improvements after nrTMS interventions has been validated [13]. NrTMS is widely used for motor recovery in stroke patients [14, 15, 16], a condition characterized by acute onset that complicates brain network remodeling [17]. Contrary, low‐grade gliomas are progressive, allowing ample time for network reorganization [18]. After glioma resection, the brain undergoes secondary network remodeling [19]. Given the continuous plasticity observed in patients with tumors, the mechanisms underlying nrTMS‐induced functional recovery may differ significantly and could potentially be even more effective.

In this study, we initially reviewed glioma cases in patients with SMA syndrome to identify critical nodes within the SMN that correlate with the recovery time of SMA syndrome. We prospectively included patients with glioma with SMA syndrome who did not regain motor function until postoperative day 7. We applied nrTMS to modulate the status of these potential nodes to evaluate whether such modulation could expedite motor recovery.

## 2 Methods

### 2.1 Patients

Fifty‐six patients who underwent an awake craniotomy (AC) at Beijing Tiantan Hospital between January 2020 and January 2023 were retrospectively reviewed. The inclusion criteria were as follows: (1) newly diagnosed low‐grade glioma (2016 World Health Organization classification of tumors of the central nervous system) located in the SMA and (2) SMA syndrome (newly occurring hemiplegia within 24 h after tumor resection). The exclusion criteria were as follows: (1) midline shift, (2) preoperative motor deficits, and (3) head motion of > 1 mm or head rotation of > 1° on rs‐fMRI.

We prospectively enrolled nine adult patients with low‐grade glioma who underwent nrTMS or sham‐nrTMS therapy at the Beijing Tiantan Hospital between February 2023 and August 2024 to validate the potential therapeutic targets associated with motor recovery. These nine patients were excluded from the retrospective analysis. The inclusion criteria were as follows: (1) patients who underwent AC and had SMA syndrome; (2) hemiplegia still present on postoperative day 7; and (3) patients who underwent fMRI scans before and after nrTMS or sham‐nrTMS therapy. Written informed consent was obtained from all participants before data acquisition. This study was approved by the Institutional Review Board of Beijing Tiantan Hospital (KY‐2022‐206‐03).

### 2.2 Motor Status Evaluation

One of the authors (a neurosurgeon) assessed motor status using a muscle strength test (MRC, UK Medical Research Council scale). When patients were under inpatient care, their motor status was evaluated 24 h preoperatively and every day postoperatively until discharge. Post‐discharge, the motor status was monitored daily through video calls facilitated by the WeChat application with the help of patients' family members or through outpatient clinical services. This follow‐up continued until the motor status returned to its preoperative state or for a duration of 6 months, whichever came first, in cases where recovery was not achieved.

Recovery time was defined as the period from surgical treatment to the time in which motor function recovered to its preoperative state (assessed using the MRC scale) or 6 months (if unrecovered). Patients were classified into recovered and non‐recovered groups according to their recovery times.

### 2.3 Intraoperative Motor Mapping Procedure

All surgical treatments were performed by two neurosurgeons with expertise in motor mapping during AC. Procedures for motor mapping have been reported in previous studies (Appendix S1) [20].

### 2.4 MRI Acquisition

A 3.0‐T MRI scanner (MAGNETOM Prisma, Siemens, Germany) was used to acquire image data of T1‐3D, T2‐fluid‐attenuated inversion recovery (Flair), and rs‐fMRI images. For rs‐fMRI, all patients were scanned for 7 min with their eyes closed and asked not to think about anything. Postoperative CT was performed in 24 h to ensure that the patients did not have resection‐related ischemia (Appendix S1).

In this retrospective analysis, the time points for image acquisition were within 24 h preoperatively and on postoperative day 7. For the prospective validation phase, the time point of image acquisition was on postoperative day 7, when nrTMS or sham‐nrTMS therapy was completed.

### 2.5 Regions of Tumor Invasion

One of the authors (a neurosurgeon) manually drew tumor and residual tumor masks based on hyperintense signals from preoperative and postoperative T2‐Flair images, respectively. Subsequently, another author (a radiologist) who was good at glioma diagnosis checked the tumor masks. Tumor and residual volumes were evaluated with a volumetric method using MRIcron software (http://www.mccauslandcenter.sc.edu/mricro/mricron/).

Extent of tumor resection was calculated as follows:

$$\begin{array}{cc} \text{Extent of tumor resection}= & 1-\left(\text{Residual tumor volume}\right)/\\ & \left(\text{Tumor volume}\right) \end{array}$$

All tumor masks were normalized to the Montreal Neurological Institute 152 T2 template using FSL software.

### 2.6 rs‐fMRI Data Preprocessing

The preprocessing pipeline has been previously reported [21], Briefly, the sequence was as follows: (a) transformation to a NIFTI file, (b) removal of the first five time points, (c) slice timing, (d) realignment, (e) normalization (normalized to EPI template1), (f) smoothing (full‐width half maximum, 4 mm), (g) temporal detrending (linear detrending), (h) covariance regressing (white matter signal: with WMMask_3mm; CSF signal: with CSFMask_3mm; head motion: Friston‐ 24 parameters), and (i) temporal filtering (0.01–0.08 Hz).

The same radiologist confirmed that normalization was correct. Normalized rs‐fMRI images that mismatched the standard EPI template were excluded.

### 2.7 Selection of Regions of Interest (ROIs) and Construction of Functional Connectivity Matrices

Regions within the SMN were selected based on the brain template “BN_Atlas_274_combined” (http://www.brainnetome.org/resource/) as ROIs [22]. ROIs in the SMN invaded by gliomas were excluded. Additionally, the bilateral cingulate cortices were added to the template because they are involved in SMA syndrome recovery [2]. A seed template based on the SMN with selected ROIs was generated by “brant” software (http://atlas.brainnetome.org/download.html). In this template, each seed was defined as a sphere with a 5‐mm diameter localized on the central coordinate of each selected ROI (Tables S1 and S2).

To construct functional‐connectivity (FC) matrices, the preprocessed rs‐fMRI data were further scrubbed (interpolation strategy: linear interpolation; FD threshold, 0.5; previous time‐point number, 1; subsequent time‐point number, 2). Subsequently, the “brant” software was used to construct an FC matrix. All the FC matrices were transformed into Z‐scores.

### 2.8 Construction and Clustering of Dynamic FC Matrices

To construct dynamic FC matrices, a “DynamicBC” package was applied in the platform MATLAB (version 2014a). We set the sliding‐window pattern option for the time‐varying model (window size, 50 repetitions; overlap, 0.98), and time alignment was set as “ahead.” The template of the SMN was the same as that used for FC matrix construction. The k‐means method was used for cluster analysis using the “DynamicBC” package. The cluster strategy was set as “Sqeuclidean,” and the number of clusters was set as “estimate.”

### 2.9 Graphic Theoretical Analysis

Positive‐weighted and dynamic FC matrices were used for graphic theoretical analysis to calculate topological properties with a series of sparsity (0.15–0.40, interval 0.01) [23]. For 1FC matrices, we calculate global efficiency, local efficiency, clustering coefficient, shortest path length, transitivity, vulnerability, and fault tolerance at the global level. Moreover, we analyzed betweenness, clustering coefficient, degree centrality, efficiency, and local efficiency at the nodal level. Furthermore, for dynamic FC matrices, we calculated only the nodal efficiency to reveal the alterations.

### 2.10 Evaluation of the Shortest Distance from Tumor Resection to the Lesional‐Hemispheric Node Related to Hand Movement

Based on postoperative T1‐3D images, the shortest distance between the surgical region and the lesional‐hemispheric node related to hand movement was calculated using MRIcron software. Detailed information is provided in Appendix S1.

### 2.11 nrTMS Therapy

Prospectively enrolled patients underwent nrTMS treatment and sham‐nrTMS treatment (TMS equipment: Magstim, England; and neuro‐navigated system: ANT‐neuro, Netherlands). The stimulated target is node A4ul_L. The duration of nrTMS therapy was 7 days, beginning on postoperative day 8. Briefly, the nrTMS therapy protocol (part 2 of Appendix S1) was as follows: (1) co‐registering the nodes A4ul_L and A4ul_H (upper limb of the BA 4 area on the healthy hemisphere) from the standard T1 template to individual T1‐3D images; (2) determination of the resting movement threshold (RMT) by stimulating A4ul_H with a figure‐8 coil (No. 4150); (3) determination of the active movement threshold (AMT) by stimulating A4ul_L with a figure‐8 coil; and (4) high‐frequency therapy stimulating A4ul_L with a cooling treatment coil (No. 3910) or sham stimulated coil (No. 3950).

The parameters of high‐frequency stimulation were as follows: intensity of stimulation, AMT; frequency of stimulation,10 Hz; duration of each stimulation, 2‐s; rounds of stimulation, 20. The interstimulus interval was generated at a minimum value using the nrTMS device. Ultimately, patients received 400 pulse stimulations per day.

### 2.12 Statistical Analyses

Clinical information, FC matrices, and topological properties were compared between the recovered and unrecovered groups or between the preoperative and postoperative periods within groups using the student's *t*‐test, paired *t*‐test, chi‐square test, and Fisher's exact test, according to the type of data.

As the number of patients with right hemispheric glioma was limited, we statistically analyzed the data based on the lesions and healthy hemispheres. To analyze the differences in the FC matrices, the significance threshold after Bonferroni correction was *p* < 1.15 × 10^−4^ $\left(\frac{0.05}{C_{30}^{2}}\right)$. For comparison of global properties, the significance threshold after correction was *p* < 0.05 $\left(\frac{0.05}{1}\right),$ while that for nodal properties was *p* < 0.0017 $\left(\frac{0.05}{30}\right).$

Pearson's correlation was used to analyze the association between topological properties (global and nodal), recovery time, and *d* value (shortest distance from A4ul_L to the surgical region). For the validation patients, we only analyzed the correlation between changes in the nodal properties of A4ul_L before and after nrTMS treatment and the recovery time.

Moreover, causal mediation analysis was performed using SPSS software (v19.0; IBM) and the PROCESS v3.0 package [24] to find the potential effects of global and nodal properties mediating between *d* values (independent variable) and recovery times (dependent variable). The parameters were as follows: model number, 4; confidence interval, 95; number of bootstraps, 5000.

## 3 Results

**FIGURE 1:**
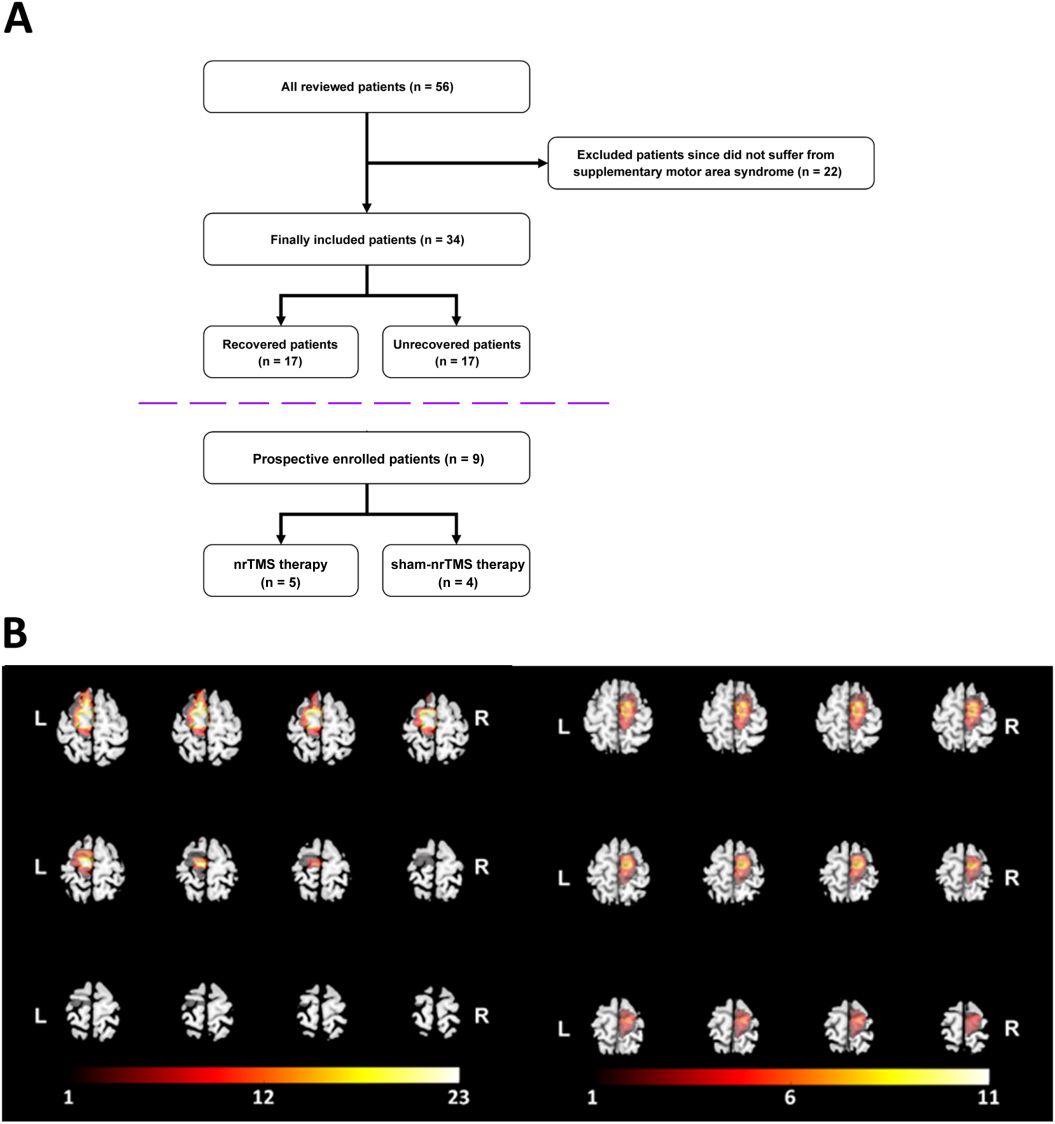
Study flow chart and tumor locations. (A) Detailed characteristics of retrospectively reviewed patients with supplementary motor area (SMA) syndrome. (B) Tumor overlapping maps of retrospective reviewed patients. The left plane indicates tumors located in the left hemisphere and the right plane indicates those in the right hemisphere. The number in the colored bar represents the number of gliomas in each location.

Finally, 34 patients were included in the retrospective analysis. Among them, 17 were classified into the recovered group, and the remaining 17 were placed in the unrecovered group (Figure 1 and Table 1). Nine patients were included in the prospective analysis. Of these, five underwent nrTMS therapy, and the remaining patients received sham‐nrTMS treatment. All patients were right‐handed.

**TABLE 1:** Clinical information between the groups.

Demographic and clinical characteristics	Recovered (*n* = 17)	Unrecovered (*n* = 17)	nrTMS therapy (*n* = 5)	Sham‐nrTMS (*n* = 4)	*p* value recovered vs. unrecovered	*p* value nrTMS vs. Sham‐nrTMS
Sex^c,d^					> 0.9999	
Male	9	9	3	1		0.5238
Female	8	8	2	3		
Age ± SEM (years)^a^	41.1 ± 3.0	37.9 ± 2.7	38.0 ± 2.6	37.0 ± 4.6	0.4456	0.8462
Education level ± SEM (y)^a^	13.8 ± 0.8	14.0 ± 0.8	15.0 ± 1.9	14.0 ± 1.2	0.8315	0.6963
Recovery time ± SEM (days)^a^	3.9 ± 0.4	28.4 ± 4.0	12.0 ± 1.6	29.5 ± 3.8	< 0.0001	0.0024
Tumor location^c,d^						
Left	11	12	3	2	> 0.9999	> 0.9999
Right	6	5	2	2		
Tumor volume ± SEM (cc)^a^	32.63 ± 2.76	26.95 ± 3.42	24.76 ± 8.66	27.01 ± 4.49	0.2047	0.8373
Extent of tumor resection ± SEM^a^	0.96 ± 0.01	0.95 ± 0.02	0.99 ± 0.01	0.89 ± 0.09	0.7161	0.2324
IDH status^b,d^					0.6562	—
Mutation	13	15	5	4		
Wildtype	4	2	0	0		
Chromosome 1p/19q status^c,d^					> 0.9999	> 0.9999
Co‐deletion	7	8	3	2		
Intact	10	9	2	2		
Histopathology^c,d^					> 0.9999	
Astrocytoma	10	9	2	2		> 0.9999
Oligodendroglioma	7	8	3	2		
Preoperative symptoms^c,d^						
Glioma‐related epilepsy (generalized)	10	12	3	1	0.7207	0.5238
Incidental	7	5	2	3		
Postoperative ischemia	0	0	0	0	—	—
Motor status on postoperative day 7^d^						
Muscle grade of upper limb < 3	0	14	5	3	—	0.5921
Muscle grade of lower limb < 3	0	8	2	3		

*Note:* Statistical analyses: ^a^Students *t*‐test; ^b^Fishers exact test between recovered and unrecovered groups; ^c^Chi‐square test between recovered and unrecovered groups; ^d^Fishers exact test between nrTMS therapy and sham‐nrTMS groups.

Abbreviations: IDH, isocitrate dehydrogenase; nrTMS, neuro‐navigated repetitive transcranial magnetic stimulation; SEM, standard error of the mean. Recovered group meant that motor function of SMA syndrome patients recovered within postoperative 7 days, and the unrecovered group meant did not recover.

Compared to the unrecovered group (28.4 ± 4.0 days), the recovery time of patients in the recovered group (3.9 ± 0.4 days) was shorter (*p* < 0.0001). Similarly, the nrTMS therapy group recovered more quickly than the sham‐nrTMS group (29.5 ± 3.8 days, *p* = 0.0024).

### 3.1 FC Differences

Regarding patients in the recovered group, only one edge that connected node A6m_H (healthy‐hemispheric medial Brodmann area (BA) 6) to node A2_L (lesional‐hemispheric BA 2) significantly increased the FC postoperatively after Bonferroni correction compared with that preoperatively (*p* < 0.0001; Table S3).

### 3.2 Characteristics of Dynamic FC Matrices

**FIGURE 2:**
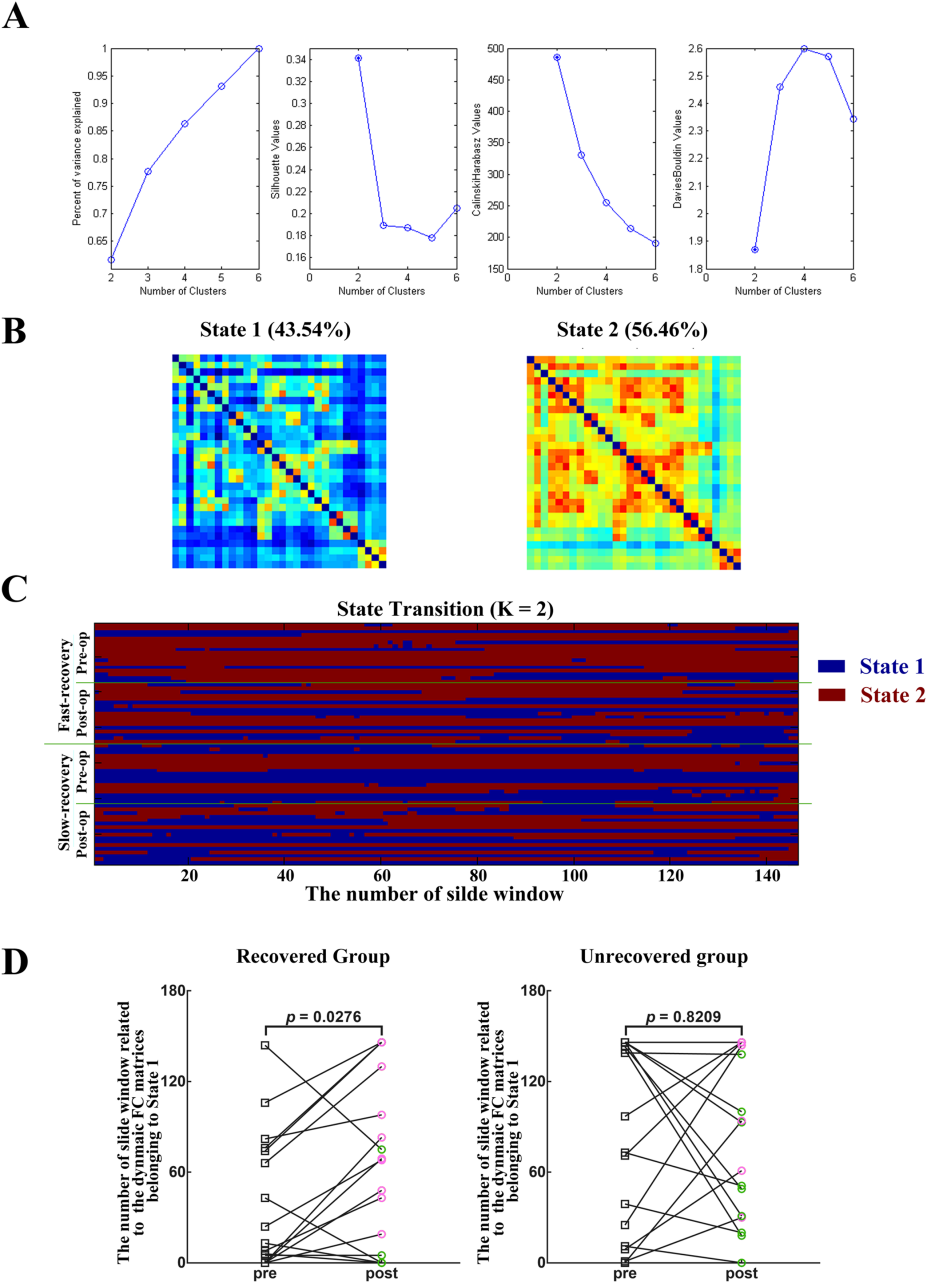
Dynamic functional‐connectivity (FC) matrix clustering. (A) Optimal number of dynamic FC matrix clusters. (B) Clustering results. All dynamic FC matrices were clustered into two states. (C) The number of slide windows related to dynamic FC matrices for all patients (blue: State 1; red: State 2). The *x*‐axis represents the number of slide windows. (D) Variations in the number of slide windows related to the dynamic FC matrices belonging to state 1 in the recovered and unrecovered groups before and after tumor resection. Green circles represent decreases and pink circles represent increases between pre‐ and postoperative numbers. Statistical analysis was performed using a paired *t*‐test.

Two FC states were clustered when all FC dynamic matrices were analyzed (Figure 2). Regarding duration period, the number of FC matrices belonging to state 1 increased significantly in the recovered group after surgery compared to preoperative values (preoperative, 38.1 ± 10.6; postoperative, 78.4 ± 14.7; *p* = 0.0276). Fewer preoperative FC matrices belonging to state 1 were observed in the recovered group (38.1 ± 10.6) than in the unrecovered group (63.3 ± 13.0; *p* = 0.0383). Furthermore, the preoperative mean dwell time of state 2 was longer (78.9 ± 13.0) in the recovered group than in the unrecovered group (35.7 ± 11.4; *p* = 0.0210, Table S4).

### 3.3 Characteristics of Global Topological Properties

Postoperative global efficiency (0.360 ± 0.009 vs. 0.306 ± 0.016; *p* = 0.0073), local efficiency (0.517 ± 0.018 vs. 0.434 ± 0.031; *p* = 0.0301), transitivity (0.540 ± 0.029 vs. 0.439 ± 0.038; *p* = 0.0460), and fault tolerance (0.845 ± 0.036 vs. 1.176 ± 0.121; *p* = 0.0161) were higher in the recovered group than in the unrecovered group (Table S5).

For patients in the recovered group, global efficiency (preoperative, 0.325 ± 0.013; postoperative, 0.360 ± 0.009; *p* = 0.0004), and local efficiency (preoperative, 0.476 ± 0.029; postoperative, 0.517 ± 0.018; *p* = 0.0357) increased after tumor resection.

**FIGURE 3:**
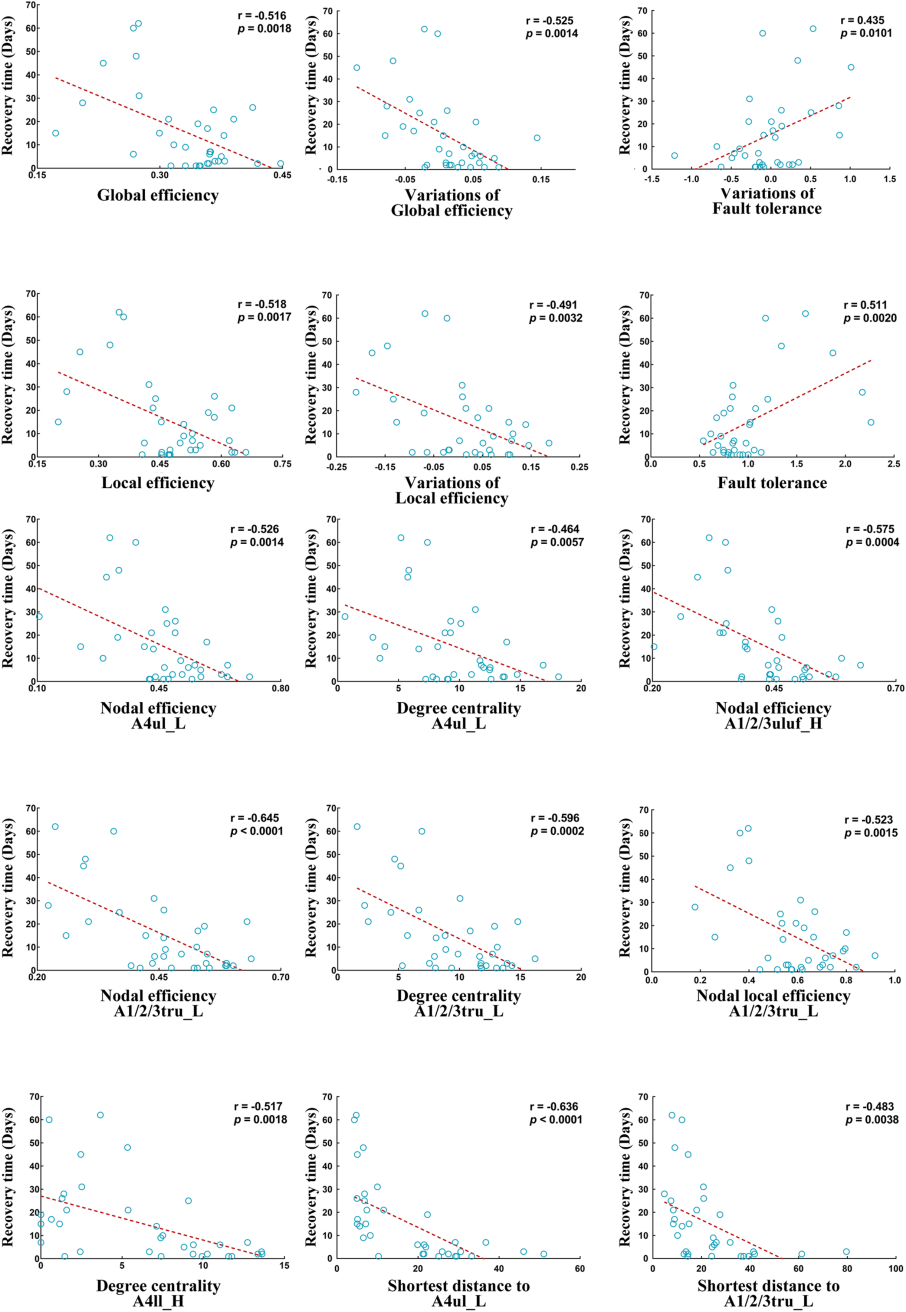
Pearson's correlation analysis. Topological properties were postoperative for both global and nodal properties. Variations in properties are defined as the postoperative properties minus the preoperative ones. A4ul_L, lesional‐hemispheric upper limb region of BA 4; A4ll_H, healthy‐hemispheric lower limb region of BA 4; A1_2_3ulhf_H, healthy‐hemispheric upper limb, head, and face region of BA 1/2/3; and A1/2/3tru_L, lesional‐hemispheric trunk region of BA 1/2/3. BA, Brodmann area.

Postoperative global efficiency (*r* = −0.516; *p* = 0.0018), local efficiency (*r* = −0.518; *p* = 0.0017), and transitivity (*r* = −0.487; *p* = 0.0035) were negatively correlated with recovery time, while postoperative fault tolerance (*r* = 0.511; *p* = 0.0020) was positively correlated with recovery time. Additionally, variations in global efficiency (*r* = −0.525; *p* = 0.0014) and local efficiency (*r* = −0.491; *p* = 0.0032) were negatively correlated with recovery time, while variations in fault tolerance (*r* = 0.435; *p* = 0.0101) were positively correlated with recovery time (Figure 3).

### 3.4 Characteristics of Nodal Properties of the Node A4ul_L

**FIGURE 4:**
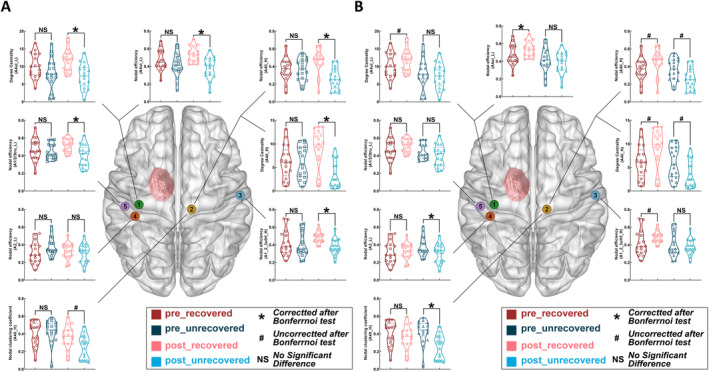
Comparisons of nodal properties. (A) Comparisons between recovered group and unrecovered group in different perioperative time. Statistical analysis with two‐sample *t*‐test. (B) Comparisons between pre‐operation and postoperation in different groups. Statistical analysis with paired *t*‐test. Red region was the example of glioma location. Green node (No. 1) = A4ul_L, lesional‐hemispheric upper limb region of BA 4; yellow node (No. 2) = A4ll_H, healthy‐hemispheric lower limb region of BA 4; blue node (No. 3) = A1_2_3ulhf_H, healthy‐hemispheric upper limb, head and face region of BA 1/2/3; orange node (No. 4) = A2_L, lesional‐hemispheric BA 2; and purple node (No. 5) = A1/2/3tru_L, lesional‐hemispheric trunk region of BA 1/2/3. BA, Brodmann area.

Regarding node A4ul_L (lesional‐hemispheric upper limb of BA 4), postoperative nodal efficiency was significantly higher in the recovered group (0.535 ± 0.020) than in the unrecovered group (0.387 ± 0.028, *p* = 0.0002), and postoperative degree centrality was higher in the recovered group than in the unrecovered group (7.305 ± 0.827, *p* = 0.0004). Furthermore, in the recovered group, nodal efficiency increased significantly postoperatively (0.472 ± 0.027) compared to preoperative values (0.535 ± 0.020; *p* = 0.0006). In the unrecovered group, the postoperative standard mean error of nodal efficiency of node A4ul_L (4.369 ± 0.773 × 10^−4^) was significantly higher than preoperatively (2.094 ± 0.445 × 10^−4^; *p* = 0.0016), based on dynamic FC matrices. In all cases, these comparisons were significant after Bonferroni correction (Tables S6–S11 and Figure 4).

In the correlation analysis, postoperative nodal efficiency (*r* = −0.464; *p* = 0.0057) and degree centrality (*r* = −0.526; *p* = 0.0014) were negatively correlated with recovery time. Moreover, the shortest distance from the surgical region to nodes A4ul_L and *d*_*A4ul_L*_ (*r* = −0.639; *p* < 0.0001) was negatively correlated with recovery time.

In the causal mediation analysis, the postoperative nodal efficiency of node A4ul_L was a mediating factor between *d*_*A4ul_L*_ and recovery time (total effect, −0.8437; direct effect, −0.6356, 75.33%; indirect effect, −0.2081, 24.67%, Table S12).

### 3.5 Characteristics of Nodal Efficiency of Node A4ul_L in the nrTMS Group

**FIGURE 5:**
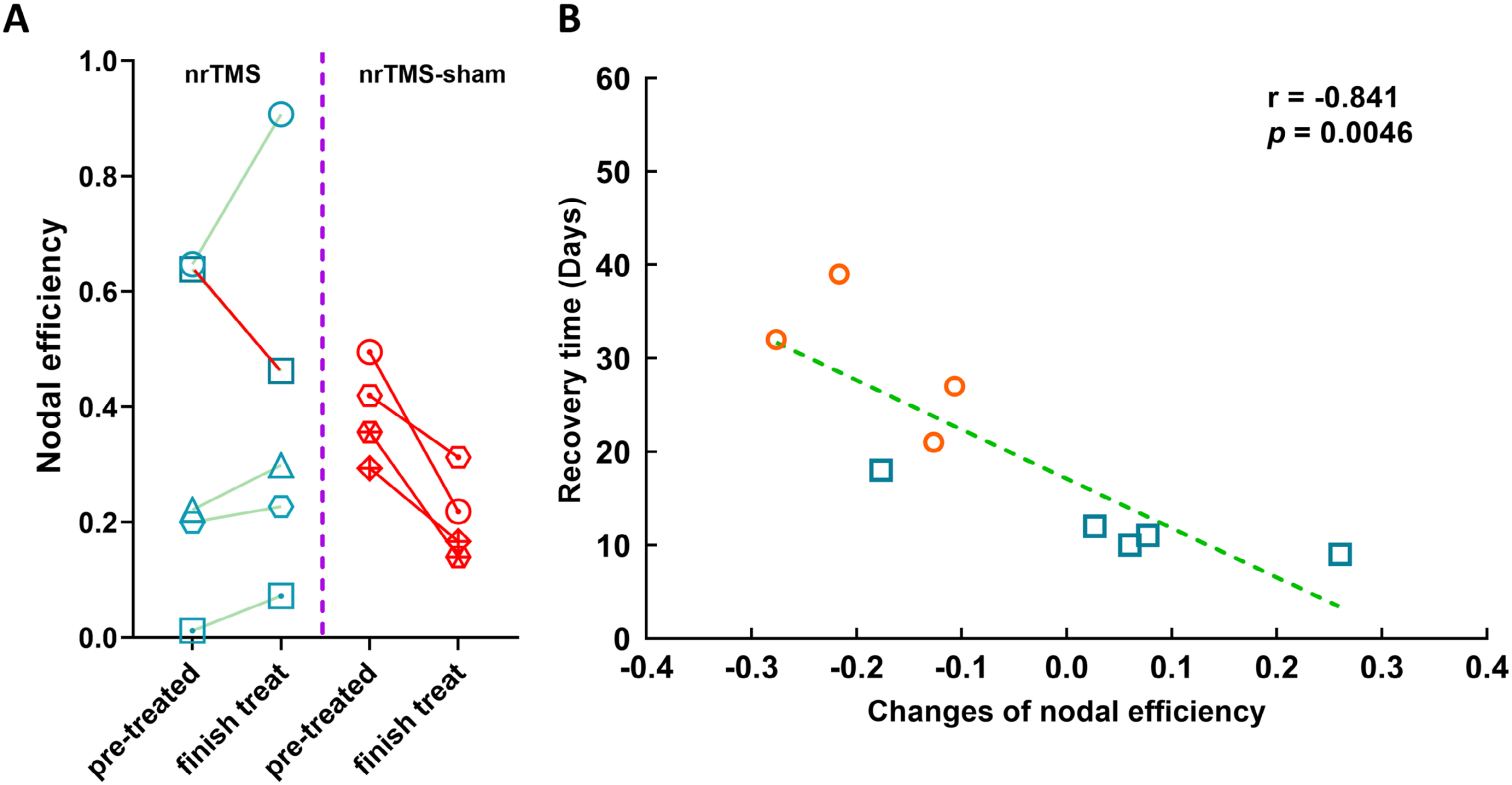
Nodal efficiency in the nrTMS therapy and sham‐nrTMS groups. (A) Variations in nodal efficiency of lesional‐hemispheric upper limb region of BA 4 (A4ul_L) in the nrTMS and sham‐nrTMS groups. Variations in properties were defined as the properties after nrTMS or sham‐nrTMS therapy minus those from before. Each symbol represents a different patient; green indicates increases and red indicates a decrease in efficiency between pre‐ and post‐therapy. (B) Correlation between variations in A4ul_L nodal efficiency and recovery time. Blue squares represent nrTMS therapy group and orange circles represent sham‐nrTMS group. BA, Brodmann area; nrTMS, neuro‐navigated repetitive transcranial magnetic stimulation.

**FIGURE 6:**
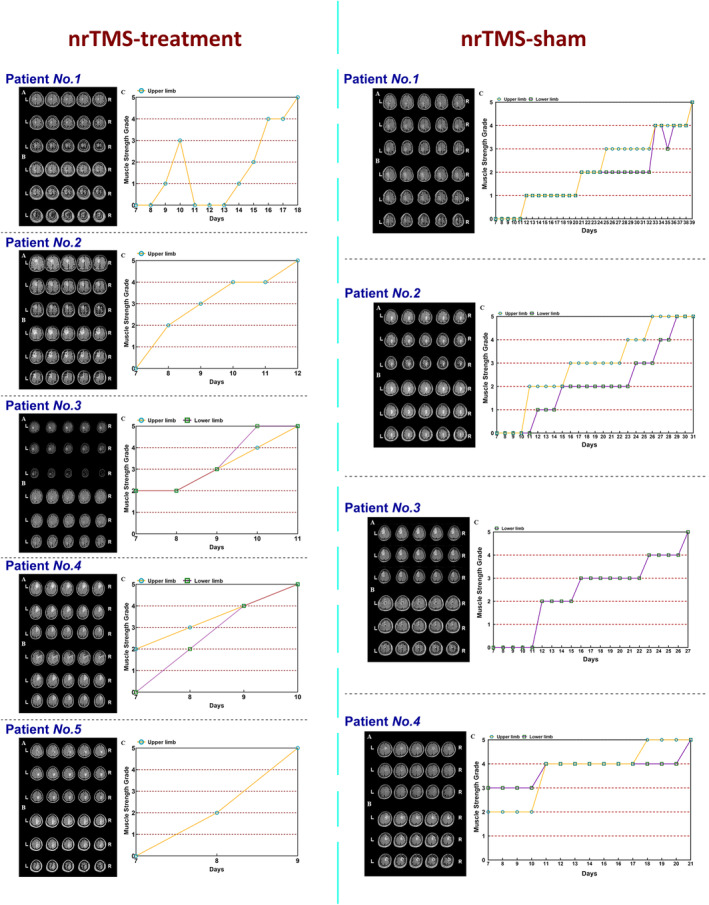
Details of the patients who underwent nrTMS therapy and sham‐nrTMS. The yellow line represents the recovery of upper limb; the purple line represents the recovery of lower limb. The muscle strength grade was evaluated using the UK Medical Research Council scale. nrTMS, neuro‐navigated repetitive transcranial magnetic stimulation.

Of the five patients in the nrTMS group, the nodal efficiency of node A4ul_L increased after nrTMS therapy in four, while no increase was observed in one. Moreover, all four patients in the sham‐nrTMS group showed decreased nodal efficiency. Additionally, the variations in nodal efficiency of node A4ul_L in the nrTMS and sham‐nrTMS groups were negatively correlated with recovery time (*r* = −0.841; *p* = 0.0046, Figures 5 and 6).

## 4 Discussion

In this retrospective analysis, we identified a notable enhancement in the nodal transmission capacity of hand motor‐related nodes within the lesional hemisphere, which was significantly correlated with rapid motor function recovery occurring within the first week after tumor resection in the SMA. Furthermore, our prospective findings suggest that high‐frequency nrTMS stimulation elevates the nodal efficiency of these hand‐motor‐related nodes. The observed increase in nodal efficiency of hand‐motor‐related nodes was positively linked to the duration of recovery.

Global efficiency, local efficiency, transitivity, and fault tolerance are important for motor recovery in patients with SMA. Global and local efficiencies represent the conveying abilities of the entire network [25]. Transitivity is the ratio of nodes that construct a triangle, representing the lowest cost of conveying information because the shortest path between these nodes is one [26]. The fault tolerance represents the maximum number of nodes that can be damaged without changing the conveying efficiency [27]. Our results showed that postoperative global efficiency, local efficiency, and transitivity were higher and fault tolerance was lower in the recovered group than in the unrecovered group. Moreover, these properties were significantly correlated with recovery time. Consequently, patients recovered motor function quickly, which was related to improved conveying ability; however, the stability of the SMN was sacrificed postoperatively.

The postoperative disparities in FC between the non‐recovered and recovered groups are consistent with the previous study [28] suggesting brain plasticity triggers functional network remodeling, thus promoting motor recovery [29, 30] in the early postoperative period. However, this interpretation warrants caution, as there are two plausible explanations for the observed differences. First, variations in FC could be the underlying cause or explanation for the different recovery times. Second, the differences may stem from the inherent comparison between individuals who successfully recovered from the syndrome and those who did not.

### 4.1 A4ul_L Is a Potential Target to Improve SMA Syndrome Recovery

Previous research has indicated that tumor resection can affect the A4ul_L region, and higher preoperative nodal efficiency in A4ul_L has been hypothesized to be correlated with the development of SMA syndrome [10]. However, our findings revealed no significant preoperative differences between the patients who recovered and those who did not. Consequently, these preoperative attributes show limited predictive power for the rate of motor recovery in patients with SMA syndrome. To more accurately forecast the speed of motor recovery in these individuals, it is imperative to focus on the postoperative characteristics or dynamic changes between the preoperative and postoperative states.

In this study, patients in the recovery group showed significantly higher postoperative nodal efficiency in the A4ul_L region than those in the non‐recovery group. Nodal efficiency is the capacity of a node to effectively transmit information within a network. Specifically, a node with high nodal efficiency plays a crucial role in facilitating the information flow [9]. A negative correlation was observed between nodal efficiency and recovery time. As mentioned above, several interpretations are possible. One of these might be that higher nodal efficiency is causally associated with rapid recovery.

In line with this interpretation, *d*_A4ul_L_ negatively affected recovery from postoperative SMA syndrome by influencing the information‐conveying abilities of the A4ul_L node, particularly through nodal efficiency [9]. Our findings further indicate that *d*_A4ul_L_ negatively affects recovery time, in part by altering the nodal efficiency of A4ul_L. These results highlight the importance of the upper limb region of BA 4 in the lesional hemisphere as a critical node for recovery from SMA syndrome. Additionally, these findings indirectly support the notion that recovery time from SMA syndrome can be predicted based on the activation patterns of the hand motor‐related areas in the lesional hemisphere [5].

Dynamic FC matrices revealed that the postoperative standard mean error of nodal efficiency for A4ul_L was notably higher in the non‐recovery group than in the recovery group. This discrepancy suggests that the conveying ability of A4ul_L in the unrecovered group was markedly unstable. This instability in conveying ability can disrupt the execution of normal motor functions, leading to motor deficits [31].

### 4.2 Application of nrTMS for Patient Rehabilitation after Glioma Resection

NrTMS, a common and non‐invasive technique, has been shown to be effective in the treatment of stroke‐related motor dysfunction [11, 32]. Recently, its application has been expanded to the rehabilitation of postoperative motor deficits resulting from gliomas. Despite its potential, this approach has encountered complexities due to several factors.

First, the application of nrTMS in glioma patients faces a significant challenge due to the absence of effective targeting strategies [33, 34]. This discrepancy is probably because of different patterns of brain network reorganization induced by stroke and glioma. Unlike strokes, low‐grade gliomas have a longer duration of network reorganization [35, 36], and subsequent tumor resection can be viewed as a secondary disruption of an already altered network. Consequently, complete reliance on previous findings regarding nrTMS therapy for stroke‐related motor deficits is impractical for patients with gliomas. For example, Ille et al. applied nrTMS to improve motor recovery in patients with acute subcortical ischemia after glioma resection. However, their study population was limited to a specific subset of patients who exhibited characteristics similar to stroke [37].

Second, the incidence of postoperative motor and language deficits varied significantly between patients with stroke and those with gliomas. With the advancements in surgical techniques, the occurrence of postoperative motor and language deficits has decreased progressively [19]. As a result, the pool of glioma patients who could potentially benefit from nrTMS was relatively small.

### 4.3 High‐Frequency Stimulation of A4ul‐L Accelerated Motor Recovery

In a previous case report on motor rehabilitation [38], a patient with a left frontal glioma underwent a contralesional‐hemispheric inhibition protocol similar to that described by Ille et al. [37]. However, our current study diverged by stimulating the lesional‐hemispheric hand motor‐related cortex with high‐frequency stimulation rather than the healthy‐hemispheric hand motor‐related cortex with low‐frequency stimulation. This decision was based on our retrospective analysis, which revealed that improving the nodal efficiency of A4ul_L was conducive to motor recovery.

High‐frequency stimulation has been shown to increase cortical excitability, thus increasing nodal efficiency [39, 40]. Our findings revealed that the recovery time in the therapy group was significantly shorter than in the sham‐nrTMS group. Notably, nrTMS therapy led to an increase in the nodal efficiency of A4ul_L in four patients, facilitating rapid motor function recovery. Contrary to this, one patient did not recover in this manner, potentially due to postoperative generalized epilepsy on postoperative day 10. Furthermore, postoperative imaging indicated that node A4ul_L was affected by postoperative edema, although this did not result in landmark distortion or normalization mismatch. However, the application of nrTMS with high‐frequency stimulation resulted in an increase in the nodal efficiency of node A4ul_L, accompanied by short‐term motor status recovery. Therefore, we posit that postoperative edema does not undermine the efficacy of nrTMS. Given the strong correlation between variations in A4ul_L nodal efficiency and recovery time, we conclude that (1) A4ul_L represents a promising target for the recovery of patients with SMA syndrome, and (2) enhancing the nodal efficiency of A4ul_L is a potential mechanism through which high‐frequency nrTMS can accelerate motor recovery.

### 4.4 Safety of nrTMS Treatment in Patient Motor Recovery

In our study, one patient experienced postoperative generalized seizures after nrTMS therapy. However, we believe that nrTMS does not pose a risk of early postoperative seizures. Previous analyses have shown that nrTMS therapy is not a risk factor for epilepsy [34, 37]. Although another study indicated a positive correlation between RMT and early postoperative seizures [41], we believe that this suggests the RMT's predictive capacity for such outcomes rather than implicating nrTMS therapy as a cause of increased risk. Furthermore, we believe that the patient's history of seizures [42] and the use of motor mapping through bipolar direct cortical stimulation were the main contributors to early postoperative seizures in this case. This has been substantiated by numerous studies that have highlighted these factors as significant risk factors for early postoperative seizures [43, 44, 45]. Consequently, we conclude that nrTMS is a safe and effective modality for the rehabilitation of SMA syndrome in patients with gliomas.

Although this study offers valuable insights, it has some limitations. First, the modest sample size required a focused approach to statistical analysis, which was addressed by comparing the lesional hemisphere with that of a healthy counterpart. This strategy was used to circumvent the inherent asymmetry of brain networks, which improves the robustness of our findings. Instead of flipping the images during preprocessing, we opted to use this method to ensure a more accurate representation of the structural and functional characteristics of the brain. Second, despite enrolling nine patients, the limited sample size remains a constraint. Consequently, our conclusion that the A4ul_L region has potential as a target for facilitating motor recovery is biologically plausible but requires validation through further research. To this end, we are currently conducting a prospective double‐blind randomized controlled trial to substantiate our findings. Third, the presence of tumor occupation poses a challenge, as it can distort “normal” anatomical landmarks. To mitigate this effect, we excluded regions near the tumor. Additionally, after normalizing the rs‐fMRI data to a standard brain template, a radiologist, who was also the author of this study, meticulously verified all data to ensure accuracy. Finally, extending the scanning time yielded more stable results. However, given the limited cooperation of patients who had recently undergone glioma resection, we had to balance the need for high‐quality images with the patient's ability to tolerate extended scanning sessions. Consequently, the scanning time in this study was determined by carefully considering both image quality and patient comfort.

## 5 Conclusions

Increased nodal efficiency in the hand‐motor cortex of the lesional hemisphere (A4ul_L) may facilitate recovery from the SMA syndrome. Therefore, A4ul_L may be a potential therapeutic target for SMA syndrome.

## Author Contributions

Study concept and design: S. Fang, S. Weng, and Z. Meng. Data acquisition and analysis: S. Fang, S. Weng, S. Li, and X. Fan. Statistics/verified analytical method: S. Weng, and Z. Meng. Writing the first draft: S. Fang, S. Weng, and Z. Meng. Supervision study: S. Fang, Y. Wang, and T. Jiang. Read and approved final version: All authors.

## Conflicts of Interest

The authors declare no conflicts of interest.

## Supporting information


Appendix S1.

## Data Availability

Anonymized data are available from the corresponding author upon reasonable request.
