# Supplementary material for: Postoperative Nodal Efficiency of the Lesional‐Hemispheric Hand Motor Area Increasing Potentially Facilitated Motor Recovery for SMA Syndrome
Source: CNS Neurosci Ther. 2024 Nov 12;30(11):e70112. doi: 10.1111/cns.70112 (PMC11557441; doi:10.1111/cns.70112)
Supplement: Supplementary file 1 — Appendix S1. [file CNS-30-e70112-s001.docx]

## Supplementary Materials

## Part 1. Intraoperative motor mapping procedure

The procedure of motor and sensory mapping was performed by one of the authors with more than 15 years of experience in functions mapping. The Ojemann stimulators (Radionics, Burlington, Massachusetts) were used to mapping motor eloquent areas (intensity 1–6 mA, square wave, frequency 60 Hz, and duration 1 s). Patient was requested to lay on the side and put the hand in rest position (palm upwards). The current of stimulation began at 1.0 mA and gradually increasing (0.5 mA each time). Threshold of stimulation was determined that the current which was induced the first positive motor reaction. The fixed sequence of motor and sensory mapping was that we firstly performed motor mapping on the precentral gyrus and secondly performed sensory mapping on the postcentral gyrus.

Regarding motor mapping, the positive reactions were defined as stimulators induced contralateral fingers or wrist unconsciously flexed or extended, or the corner of mouth unconsciously twitching. Regarding sensory mapping, the positive reaction was defined as stimulators induced contralateral fingers, palm, wrist, or lips feeling numbness (like electrical sensation). Each site was stimulated three times. If more than twice stimulations induced positive reactions, the site was defined as positive. The positive sites were subsequently labeled by sterile markers with diameters of 5 mm.

## Part 2. Transcranial magnetic stimulation therapy

A neuro-navigation repetitive transcranial magnetic stimulation (nrTMS; TMS equipment: Magstim^®^, England; and neuro-navigated system: ANT-neuro^®^, Netherland) was implemented on the prospectively enrolled patients. The stimulated target was the node A4ul_L. The period of nrTMS therapy or sham-nrTMS was 7 days at the beginning of the postoperative 8^th^ days. The nrTMS therapy or sham-nrTMS protocol consisted of the following steps:

1. *Co-registering the nodes of A4ul_L and A4ul_H (upper limb of BA 4 area on the healthy hemisphere) from the standard T1 template to individual T1-3D images*

With the assistance of the co-registration and normalization functions in the SPM 12 software, the masks (A4ul_L and A4ul_H) that were based on the standard MNI template, was co-registered to the individual T1-3D template. Consequently, we were able to manually identify the locations of the A4ul_L and A4ul_H nodes in the individual T1-3D images within the neuronavigation system of nrTMS, using the results displayed on the MRIcron software as a reference.

2. *Determination of resting movement threshold (rMT) on the healthy hemisphere*

A figure-8 coil (No. 4150) was used for stimulation on the mask of the node of A4ul_H. Electromyography (EMG) was employed as a monitor, and the stimulation began at 50% stimulating intensity. The stimulating intensity was increased by 5% with each time until the EMG amplitude of the abductor pollicis brevis muscle exceeded 50 *uV* in at least 5 out of 10 consecutive stimulations.

1) If the EMG amplitude exceeded 50 *uV* in exactly 5 out of 10 consecutive stimulations, the stimulation intensity at that point was defined as the rMT. However, if the EMG amplitude exceeded 50 *uV* more than 5 out of 10 consecutive stimulations, the stimulation intensity at that point was denoted as ‘n%’ and needed to be reduced.

2) The procedure was repeated by decreasing the intensity by 3% (n-3%). If the EMG amplitude exceeded 50 *uV* in exactly 5 out of 10 consecutive stimulations, the rMT was defined as n-3%. If more than 5 times, the intensity continued decreased to n-4%, and if less than 5 times, the intensity should increase to n-1%

3) When the intensity was set as n-4%, if the EMG amplitude exceeded 50 *uV* in less than 5 out of 10 stimulations, the rMT was set as n-3%. Otherwise, the rMT was set as n-4%.

4) When the intensity was set as n-1%, if the EMG amplitude exceeded 50 *uV* in less than 5 out of 10 stimulations, the rMT was defined as n%. Otherwise, the intensity continued decreased to n-2%.

5) When the intensity was set as n-2%, if the EMG amplitude fell below 50 *uV* in less than 5 out of 10 stimulations, the rMT was set as n-1%. Otherwise, the rMT was set as n-2%.

2. *Determination of active movement threshold (AMT) on the lesioned hemisphere*

The figure-8 coil (No. 4150) was used for stimulation on A4ul_L, starting at 100% of the RMT and increasing at 5% in each step until the MEP evoke was higher than 50 *uV* in more than 5 out of 10 stimulations or the intensity of AMT achieved to 150% RMT.

3. *High-frequency stimulating therapy*

High-frequency stimulation was administered using a cooling treatment coil (No. 3910) and sham-nrTMS coil (No. 3950) on the A4ul_L. The stimulation parameters included: the intensity of stimulation: AMT; frequency of stimulation: 10 Hz; duration of each stimulation: 2 seconds; and rounds of stimulation: 20. The interstimulus interval was generated by the TMS device at the minimum value.

## Part 3. Information of patients who underwent nrTMS therapy and sham-nrTMS

- *nrTMS therapy group*

*Patient No. 1*.

**
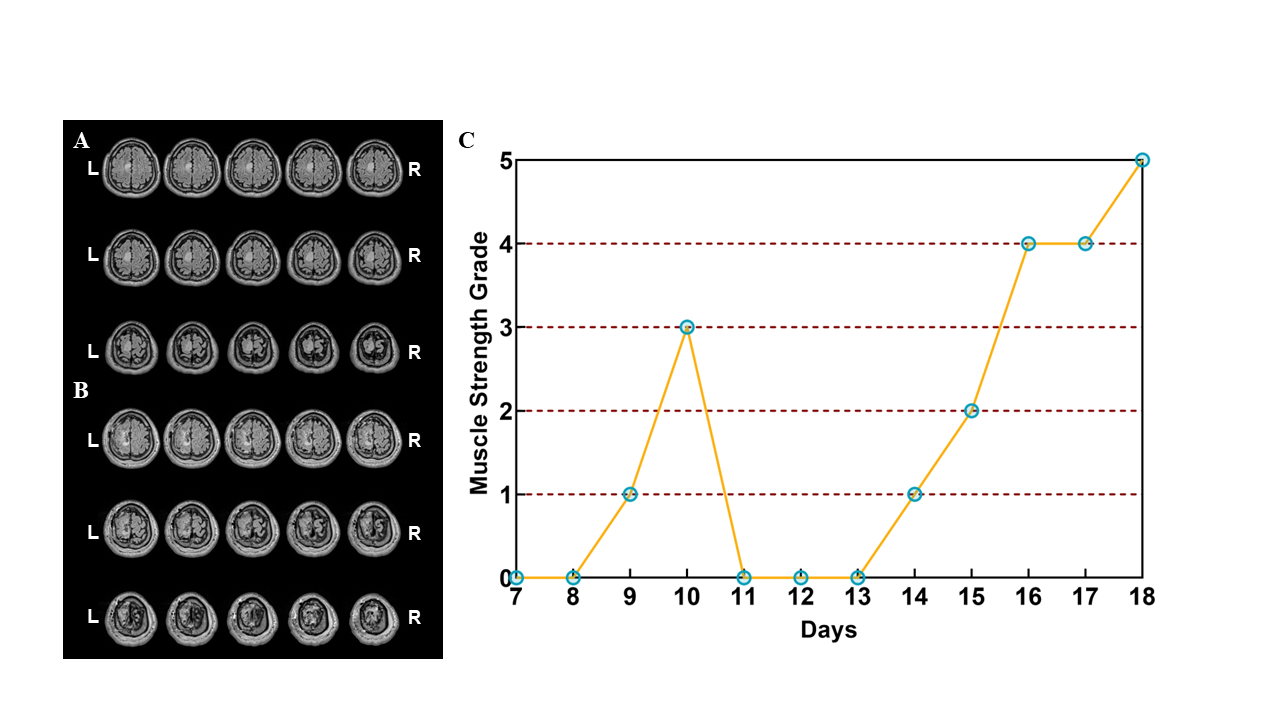
** The chief complaint of *patient No.1* was generalized glioma-related epilepsy. His tumor volume was 4.61cc, the extent of tumor resection was 1.00, and preoperative KPS score was 100. He underwent awaken craniotomy and motor mapping before tumor resection. He experienced SMA syndrome within 24 hours after tumor resection, and his muscle strength of upper limb was grade 0-1 at 7 days in post-operation. Hence, he was enrolled and receipt nrTMS therapy. After 3 times therapy, his muscle strength of upper limb recovered to grade 4. However, he suffered from a generalized epilepsy on the day after nrTMS therapy, and his muscle strength decreased to 0. After finishing nrTMS therapy at 14 days in post-operation, he underwent rs-fMRI scan, but his muscle strength remained to be grade 0-1. At 15 days in post-operation, his muscle strength recovered to grade 2 before he discharged. Moreover, we acquired his follow-up information by WeChat video every day. At 16 days in post-operation, his muscle strength recovered to grade 4. Finally, at 18 days in post-operation, his muscle strength of upper limb recovered to grade 5. His information was shown as follow:

Tumor location, region of tumor resection, and recovery information. A) Preoperative T2-Flair image; B) Postoperative T2-Flair image; C) Motor recovery information.

*Patient No. 2*.

**
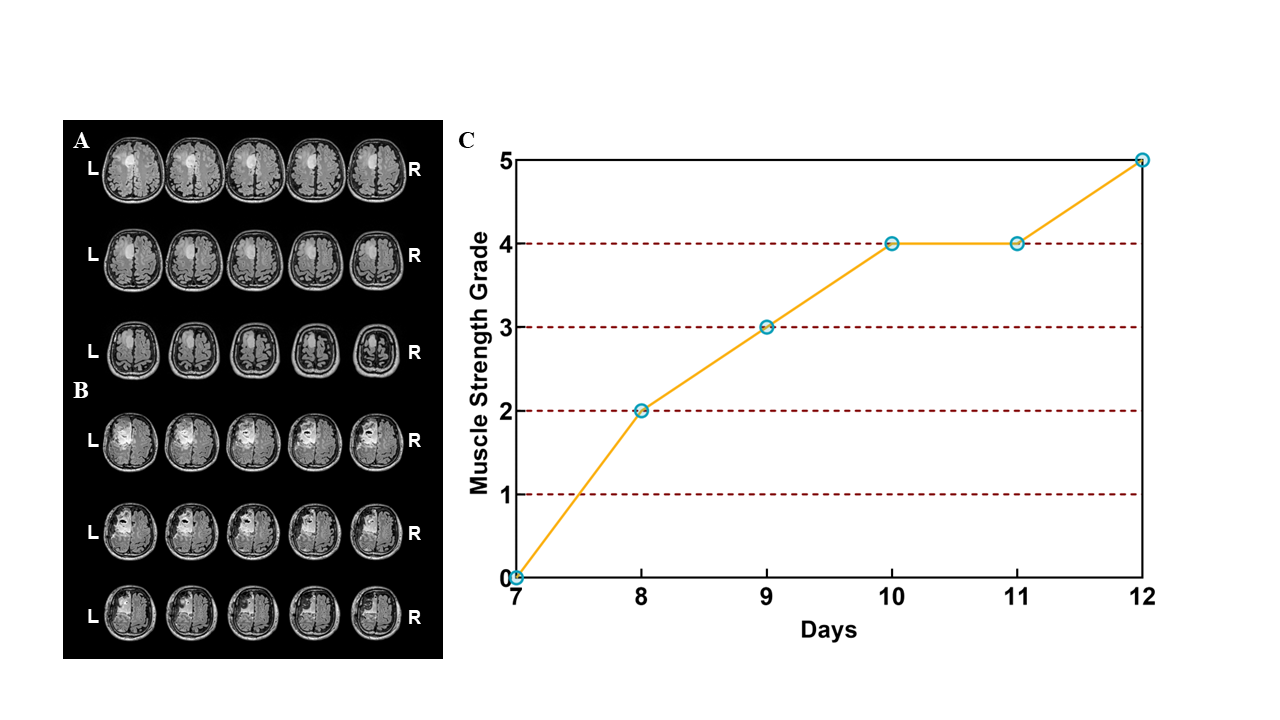
** The chief complaint of *patient No.2* was generalized glioma-related epilepsy. His tumor volume was 24.57cc, the extent of tumor resection was 1.00, and preoperative KPS score was 100. He underwent awaken craniotomy and motor mapping before tumor resection. He experienced SMA syndrome within 24 hours after tumor resection, and his muscle strength of upper limb was grade 0 at 7 days in post-operation. Hence, he was enrolled and receipt nrTMS therapy. After twice therapy, his muscle strength of upper limb recovered to grade 3. At 10 days in post-operation, his muscle strength of upper limb recovered to grade 4. Finally, at 12 days in post-operation, his muscle strength of upper limb recovered to grade 5. His information was shown as follow:

Tumor location, region of tumor resection, and recovery information. A) Preoperative T2-Flair image; B) Postoperative T2-Flair image; C) Motor recovery information.

*Patient No. 3*.

**
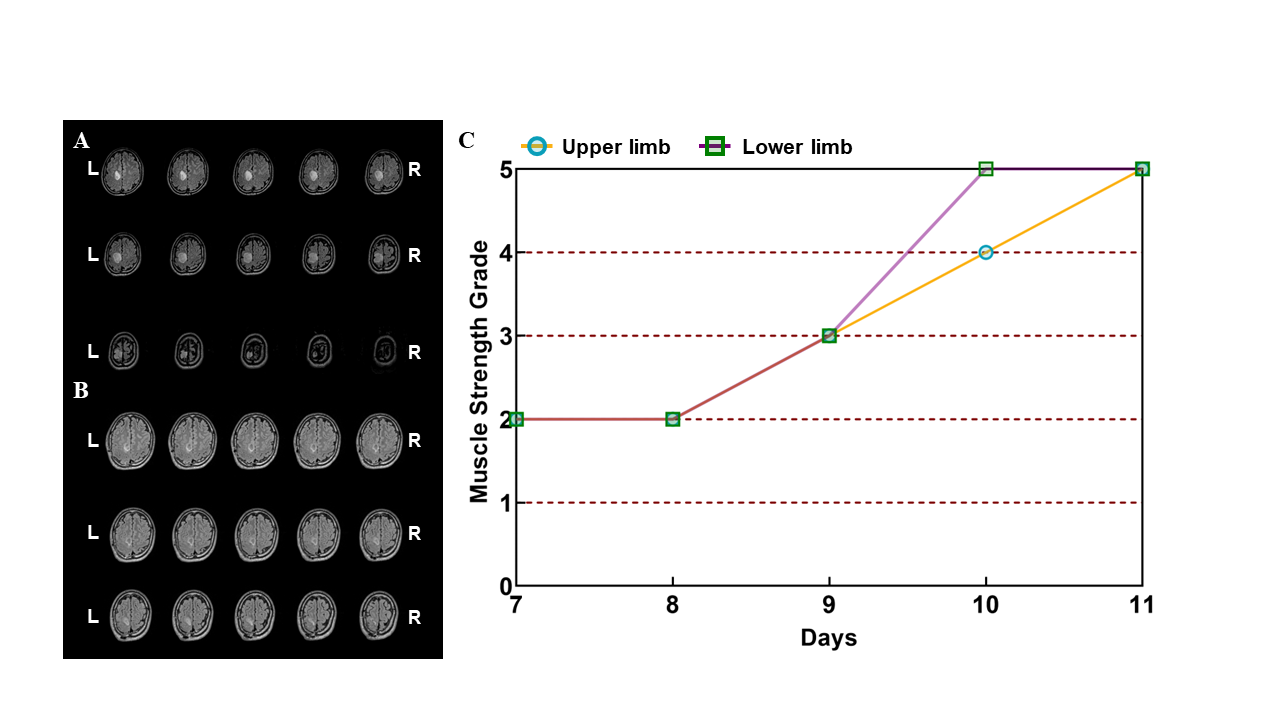
** The *patient No.3* was incidental found. The tumor volume was 23.97cc, the extent of tumor resection was 1.00, and preoperative KPS score was 100. She underwent awaken craniotomy and motor mapping before tumor resection, and experienced SMA syndrome within 24 hours after tumor resection. Her muscle strength of both upper limb and lower limb were grade 2 at 7 days in post-operation. After twice therapy, her muscle strength of upper limb and lower limb recovered to grade 3, respectively. At 10 days in post-operation, her muscle strength of upper limb recovered to grade 5- and lower limb totally recovered. Finally, at 11 days in post-operation, her muscle strength of upper limb recovered to grade 5. Her information was shown as follow:

Tumor location, region of tumor resection, and recovery information. A) Preoperative T2-Flair image; B) Postoperative T2-Flair image; C) Motor recovery information.

*Patient No. 4*.


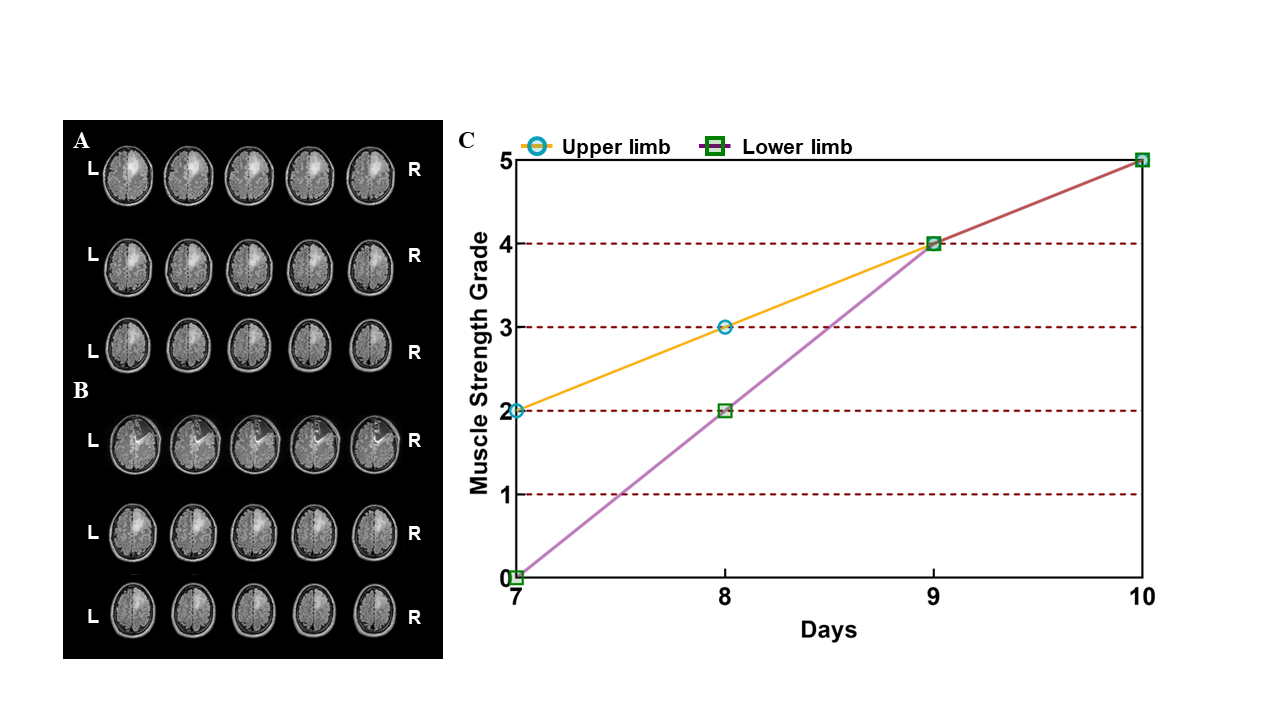
The chief complaint of *patient No.4* was generalized glioma-related epilepsy. The tumor volume was 56.16cc, the extent of tumor resection was 0.96, and preoperative KPS score was 100. She underwent awaken craniotomy and motor mapping before tumor resection, and experienced SMA syndrome within 24 hours after tumor resection. Her muscle strength of upper limb was grade 2 and lower limb was grade 0 at 7 days in post-operation. After twice times therapy, her muscle strength of upper and lower limbs recovered to grade 4. Finally, at 10 days in post-operation, her muscle strength of upper and lower limbs recovered to grade 5. Her information was shown as follow:

Tumor location, region of tumor resection, and recovery information. A) Preoperative T2-Flair image; B) Postoperative T2-Flair image; C) Motor recovery information.

*Patient No. 5*.

**
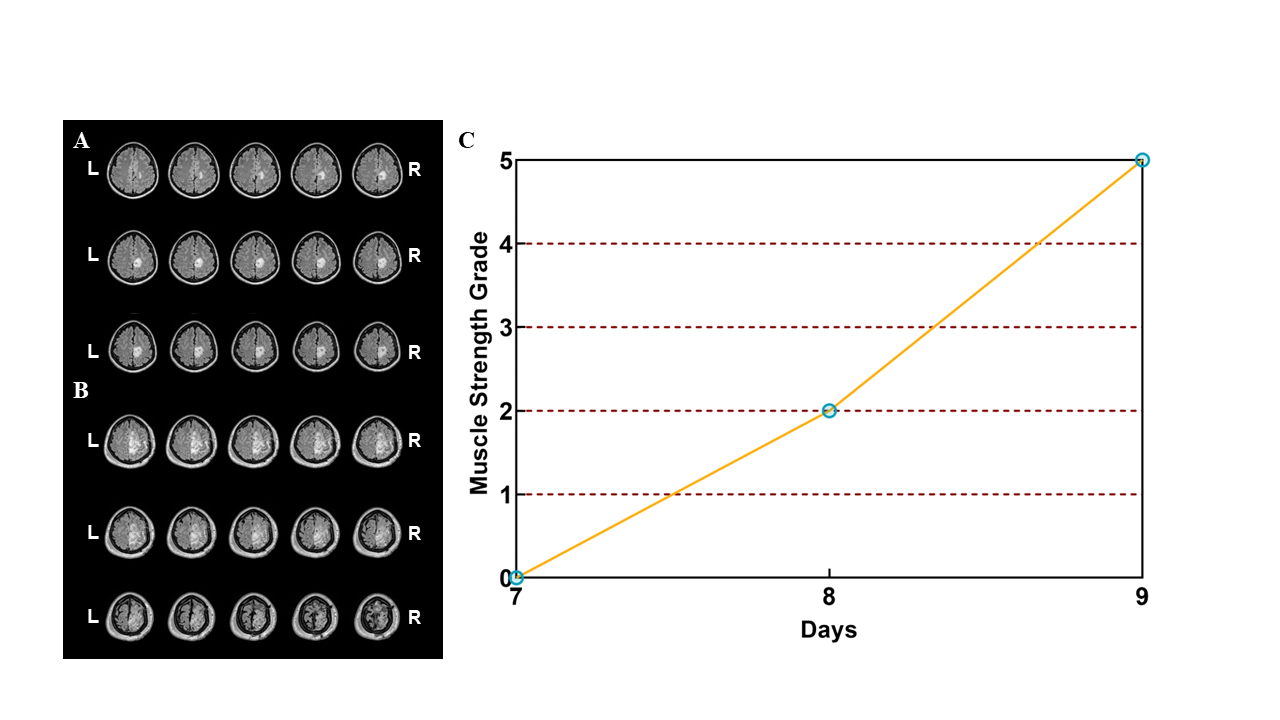
** The *patient No.5* was incidental found. The tumor volume was 14.47cc, the extent of tumor resection was 1, and preoperative KPS score was 100. He underwent awaken craniotomy and motor mapping before tumor resection, and experienced SMA syndrome within 24 hours after tumor resection. Her muscle strength of upper limb was grade 0 at 7 days in post-operation. After once times therapy, her muscle strength of upper limb recovered to grade 2. Finally, at 9 days in post-operation, her muscle strength of upper limb recovered to grade 5. Her information was shown as follow:

Tumor location, region of tumor resection, and recovery information. A) Preoperative T2-Flair image; B) Postoperative T2-Flair image; C) Motor recovery information.

- *Sham-nrTMS group*

*Patient No. 1*.


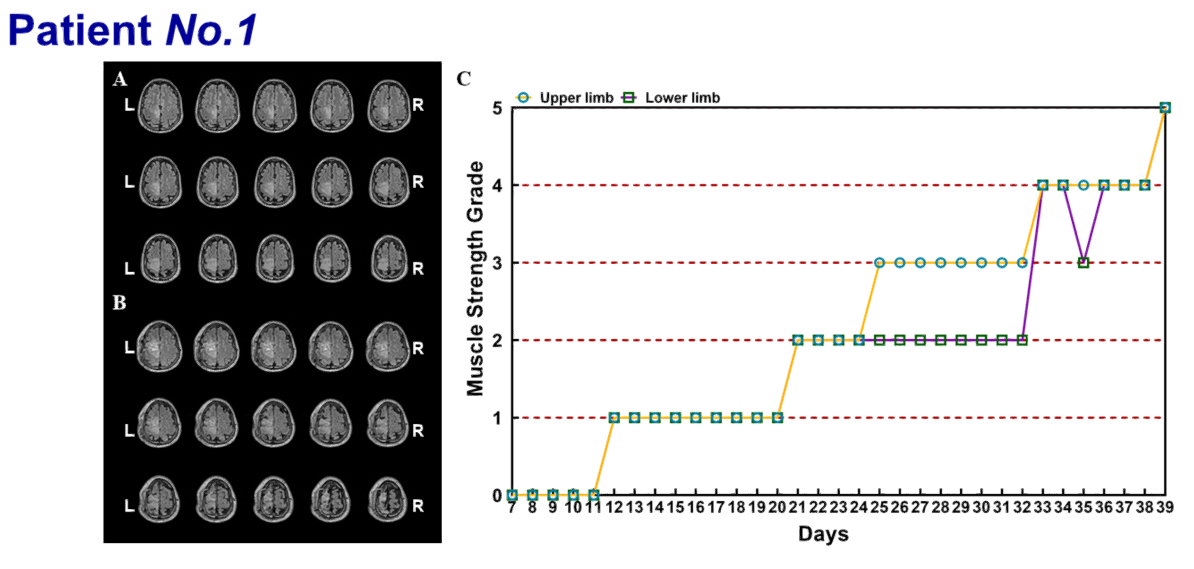
 The chief complaint of *patient No.1* was incidental. Her tumor volume was 24.50cc, the extent of tumor resection was 0.63, and preoperative KPS score was 100. She underwent awaken craniotomy and motor mapping before tumor resection. She experienced SMA syndrome within 24 hours after tumor resection, and her muscle strength of right upper and lower limbs were grade 0 at 7 days in post-operation. Hence, she was enrolled and receipt sham-nrTMS group. After 4 times sham stimulation, her muscle strength of upper and lower limbs recovered to grade 1, and her muscle strength upper and lower limbs remain at grade 1 after finishing sham-nrTMS therapy. Moreover, we acquired her follow-up information by WeChat video every day. Until to postoperative 32^th^ day, her muscle strength of the upper limb recovered to grade 3 and lower limb recover to grade 2. Finally, at 39 days in post-operation, her muscle strength of upper and lower limb recovered to grade 5. Her information was shown as follow:

Tumor location, region of tumor resection, and recovery information. A) Preoperative T2-Flair image; B) Postoperative T2-Flair image; C) Motor recovery information.

*Patient No. 2*.


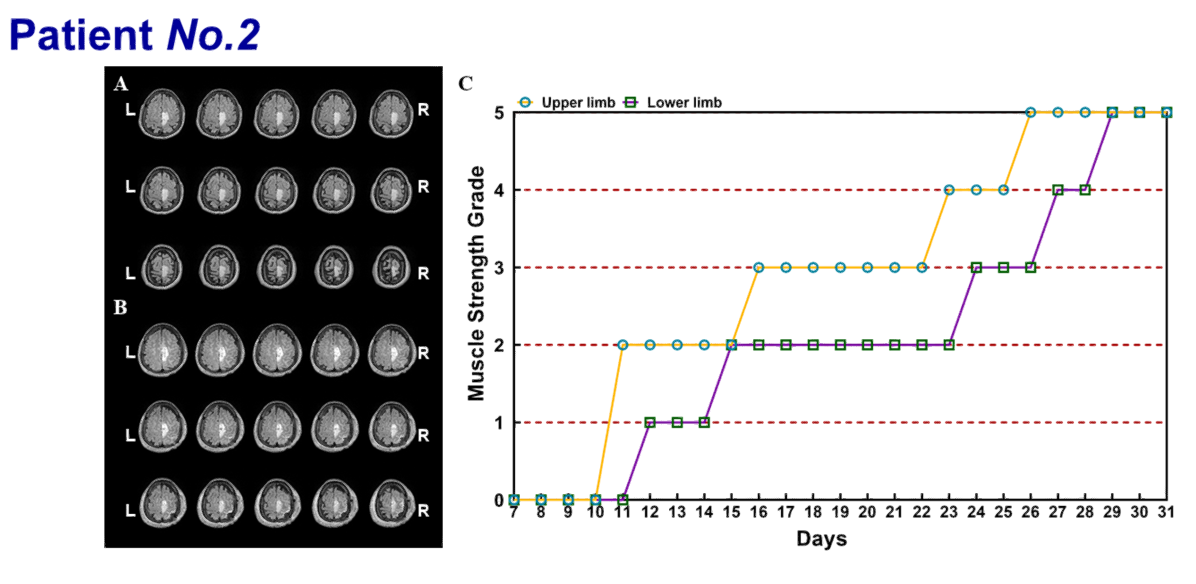
 The *patient No.2* was incidentally diagnosed. His tumor volume was 15.87cc, the extent of tumor resection was 1.00, and preoperative KPS score was 100. He underwent awaken craniotomy and motor mapping before tumor resection. He experienced SMA syndrome within 24 hours after tumor resection, and his muscle strength of upper and lower limbs was grade 0 at 7 days in post-operation. Hence, he was enrolled and receipt sham-nrTMS therapy. After 4 times therapy, his muscle strength of upper limb recovered to grade 2, and lower limb recovered to grade 1. At 15 days in post-operation, his muscle strength of upper limb recovered to grade 3, and lower limb recovered to grade 2. Finally, at 31 days in post-operation, his muscle strength of upper limb recovered to grade 5. His information was shown as follow:

Tumor location, region of tumor resection, and recovery information. A) Preoperative T2-Flair image; B) Postoperative T2-Flair image; C) Motor recovery information.

*Patient No. 3*.


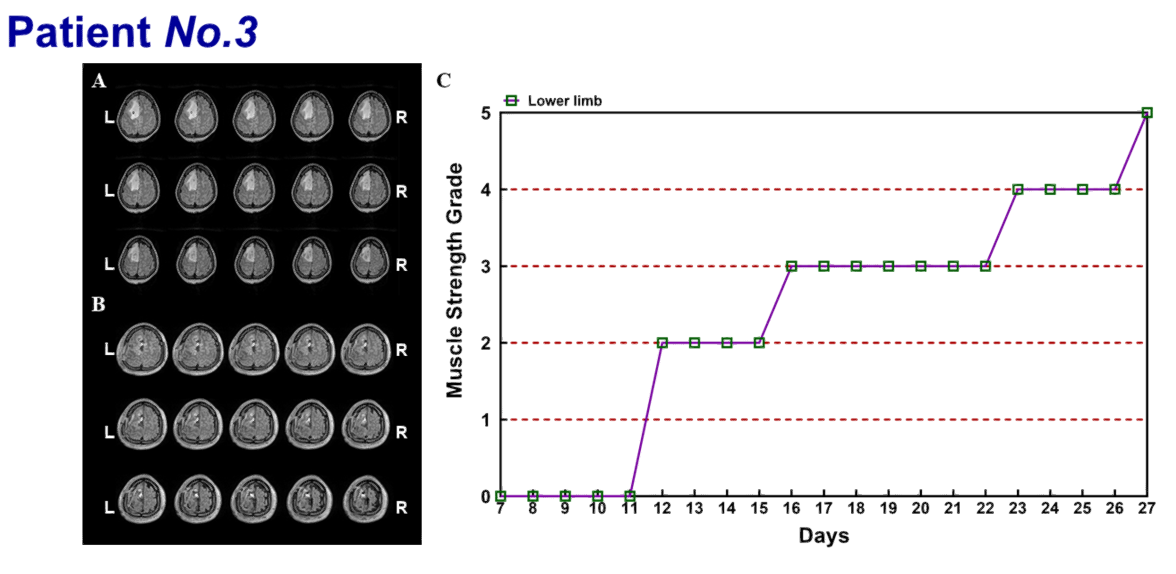
 The *patient No.3* was incidental found. The tumor volume was 36.88cc, the extent of tumor resection was 0.93, and preoperative KPS score was 100. She underwent awaken craniotomy and motor mapping before tumor resection, and experienced SMA syndrome within 24 hours after tumor resection. Her muscle strength of lower limb was grade 0 at 7 days in post-operation. After five times sham-nrTMS therapy, her muscle strength of lower limb recovered to grade 2. Finally, at 27 days in post-operation, her muscle strength of lower limb recovered to grade 5. Her information was shown as follow:

Tumor location, region of tumor resection, and recovery information. A) Preoperative T2-Flair image; B) Postoperative T2-Flair image; C) Motor recovery information.

*Patient No. 4*.


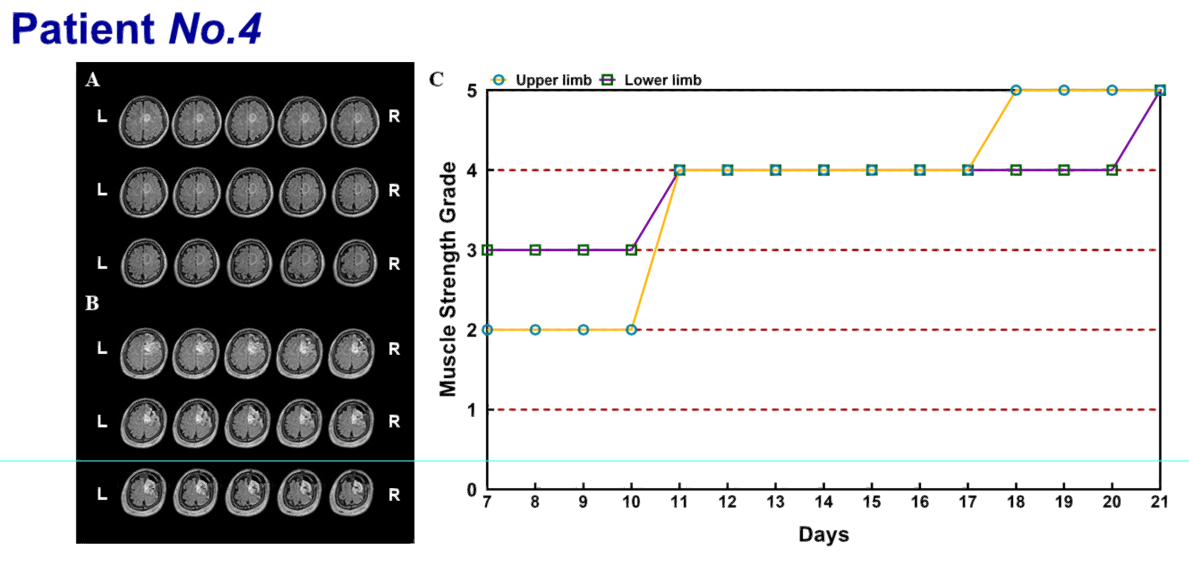
The chief complaint of *patient No.4* was generalized glioma-related epilepsy. The tumor volume was 30.79cc, the extent of tumor resection was 1, and preoperative KPS score was 100. She underwent awaken craniotomy and motor mapping before tumor resection, and experienced SMA syndrome within 24 hours after tumor resection. Her muscle strength of upper limb was grade 2 and lower limb was grade 3 at 7 days in post-operation. After four times sham-nrTMS therapy, her muscle strength of upper and lower limbs recovered to grade 4. Finally, at 21 days in post-operation, her muscle strength of upper and lower limbs recovered to grade 5. Her information was shown as follow:

Tumor location, region of tumor resection, and recovery information. A) Preoperative T2-Flair image; B) Postoperative T2-Flair image; C) Motor recovery information.

| **Table S1. Montreal Neurological Institute locations of 30 nodes in the sensorimotor network for left glioma** | | | | | | | |
| --- | --- | --- | --- | --- | --- | --- | --- |
| **Regions of interesting** | **Modified Cyto-architectonic** | **Lesional hemisphere** | | | **Healthy hemisphere** | | |
|  |  | X | Y | Z | X | Y | Z |
| A6dl_R | Dorsolateral area BA 6 | - | - | - | 20 | 4 | 64 |
| A6m_R | Medial area BA 6 | - | - | - | 7 | -4 | 60 |
| A6vl_R | Ventrolateral BA 6 | - | - | - | 34 | 8 | 54 |
| A6cdl_R | Caudal dorsolateral BA 6 | - | - | - | 33 | -7 | 57 |
| A4ul_L(R) | Area BA 4 (upper limb) | -26 | -25 | 63 | 34 | -19 | 59 |
| A4t_L(R) | Area BA 4 (trunk) | -13 | -20 | 73 | 15 | -22 | 71 |
| A4tl_L(R) | Area BA 4 (tongue and larynx) | -52 | 0 | 8 | 54 | 4 | 9 |
| A6cvl_L(R) | Caudal ventrolateral BA 6 | -49 | 5 | 30 | 51 | 7 | 30 |
| A1/2/3ll_R | Area BA 1/2/3 (lower limb) | - | - | - | 10 | -34 | 54 |
| A4ll_R | Area BA 4 (lower limb) | - | - | - | 5 | -21 | 61 |
| A1/2/3ulhf_L(R) | Area BA 1/2/3 (upper limb and face) | -50 | -16 | 43 | 50 | -14 | 44 |
| A1/2/3tonIa_L(R) | Area BA 1/2/3 (tongue and larynx) | -56 | -14 | 16 | 56 | -10 | 15 |
| A2_L(R) | Area BA 2 | -46 | -30 | 50 | 48 | -24 | 48 |
| A1/2/3tru_L(R) | Area BA 1/2/3 (trunk) | -21 | -35 | 68 | 20 | -33 | 69 |
| A24cd_ L(R) | Caudal dorsal BA 24 | -5 | 7 | 37 | 4 | 6 | 38 |
| A23c_ L(R) | caudal area BA 23 | -7 | -23 | 41 | 6 | -20 | 40 |
| MPMtha_L(R) | Pre-motor thalamus | -18 | -13 | 3 | 12 | -14 | 1 |
| Stha_L(R) | Sensory thalamus | -18 | -23 | 4 | 18 | -22 | 3 |
| *BA = Brodmann area. | | | | | | | |

## Part 4. Supplementary tables

| **Table S2. Montreal Neurological Institute locations of 30 nodes in the sensorimotor network for right glioma** | | | | | | | |
| --- | --- | --- | --- | --- | --- | --- | --- |
| **Regions of interesting** | **Modified Cyto-architectonic** | **Lesional hemisphere** | | | **Healthy hemisphere** | | |
|  |  | X | Y | Z | X | Y | Z |
| A6dl_L | Dorsolateral area BA 6 | -18 | -1 | 65 | - | - | - |
| A6m_L | Medial area BA 6 | -6 | -5 | 68 | - | - | - |
| A6vl_L | Ventrolateral BA 6 | -32 | 4 | 55 | - | - | - |
| A6cdl_L | Caudal dorsolateral BA 6 | -32 | -9 | 58 | - | - | - |
| A4ul_L(R) | Area BA 4 (upper limb) | -26 | -25 | 63 | 34 | -19 | 59 |
| A4t_L(R) | Area BA 4 (trunk) | -13 | -20 | 73 | 15 | -22 | 71 |
| A4tl_L(R) | Area BA 4 (tongue and larynx) | -52 | 0 | 8 | 54 | 4 | 9 |
| A6cvl_L(R) | Caudal ventrolateral BA 6 | -49 | 5 | 30 | 51 | 7 | 30 |
| A1/2/3ll_L | Area BA 1/2/3 (lower limb) | -8 | -38 | 58 | - | - | - |
| A4ll_L | Area BA 4 (lower limb) | -4 | -23 | 61 | - | - | - |
| A1/2/3ulhf_L(R) | Area BA 1/2/3 (upper limb and face) | -50 | -16 | 43 | 50 | -14 | 44 |
| A1/2/3tonIa_L(R) | Area BA 1/2/3 (tongue and larynx) | -56 | -14 | 16 | 56 | -10 | 15 |
| A2_L(R) | Area BA 2 | -46 | -30 | 50 | 48 | -24 | 48 |
| A1/2/3tru_L(R) | Area BA 1/2/3 (trunk) | -21 | -35 | 68 | 20 | -33 | 69 |
| A24cd_ L(R) | Caudal dorsal BA 24 | -5 | 7 | 37 | 4 | 6 | 38 |
| A23c_ L(R) | caudal area BA 23 | -7 | -23 | 41 | 6 | -20 | 40 |
| MPMtha_L(R) | Pre-motor thalamus | -18 | -13 | 3 | 12 | -14 | 1 |
| Stha_L(R) | Sensory thalamus | -18 | -23 | 4 | 18 | -22 | 3 |
| *BA = Brodmann area. | | | | | | | |

| **Table S3. Functional connectivity comparison between recovered and unrecovered groups** | | | | | | | | |
| --- | --- | --- | --- | --- | --- | --- | --- | --- |
| Edge | Preoperative | | Postoperative | | *p* value  (Two-sample t test) | | *p* value  (Paired t test) | |
|  | Recovered | Unrecovered | Recovered | Unrecovered | Preoperative | Postoperative | Recovered | Unrecovered |
|  |  |  |  |  | Recovered *vs.* Unrecovered | Recovered *vs.* Unrecovered | Preoperative *vs.* Postoperative | Preoperative *vs.* Postoperative |
| A6dl_H to A6cvl_L | 0.453 ± 0.044 | 0.612 ± -0.115 | 0.509 ± 0.044 | 0.497 ± 0.028 | 0.0044 | 0.8644 | 0.3698 | 0.0608 |
| A6m_H to A4t_L | 0.634 ± 0.068 | 0.612 ± -0.086 | 0.761 ± 0.031 | 0.526 ± 0.048 | 0.7873 | 0.0041 | 0.0626 | 0.1891 |
| A6m_H to A2_L | 0.500 ± 0.047 | 0.525 ± 0.006 | 0.609 ± 0.037 | 0.530 ± 0.050 | 0.7305 | 0.2102 | < 0.0001* | 0.9282 |
| A6m_H to A1_2_3tru_L | 0.674 ± 0.037 | 0.603 ± -0.004 | 0.786 ± 0.023 | 0.600 ± 0.053 | 0.3048 | 0.0077 | 0.0199 | 0.9584 |
| A6m_H to A24cd_L | 0.636 ± 0.056 | 0.700 ± -0.152 | 0.768 ± 0.026 | 0.548 ± 0.032 | 0.3300 | 0.0071 | 0.0092 | 0.0530 |
| A6m_H to A23c_L | 0.480 ± 0.055 | 0.492 ± -0.091 | 0.650 ± 0.027 | 0.400 ± 0.041 | 0.8693 | 0.0030 | 0.0200 | 0.2178 |
| A6cdl_H to A4ul_L | 0.609 ± 0.038 | 0.621 ± 0.022 | 0.725 ± 0.043 | 0.643 ± 0.051 | 0.8535 | 0.2012 | 0.0029 | 0.7106 |
| A6cdl_H to A23c_L | 0.398 ± 0.068 | 0.434 ± -0.070 | 0.570 ± 0.034 | 0.364 ± 0.033 | 0.6411 | 0.0076 | 0.0095 | 0.3178 |
| A4ul_L to A4ll_H | 0.684 ± 0.045 | 0.730 ± -0.042 | 0.798 ± 0.036 | 0.687 ± 0.048 | 0.5072 | 0.0862 | 0.0084 | 0.3957 |
| A4ul_L to A23c_L | 0.422 ± 0.065 | 0.462 ± -0.113 | 0.598 ± 0.030 | 0.349 ± 0.038 | 0.6038 | 0.0031 | 0.0095 | 0.1197 |
| A4ul_H to A4t_L | 0.571 ± 0.073 | 0.577 ± -0.081 | 0.726 ± 0.035 | 0.497 ± 0.056 | 0.9494 | 0.0050 | 0.0256 | 0.2361 |
| A4ul_H to A4ll_H | 0.695 ± 0.042 | 0.738 ± -0.064 | 0.813 ± 0.012 | 0.674 ± 0.025 | 0.3875 | 0.0227 | 0.0041 | 0.2918 |
| A4ul_H to A24cd_L | 0.457 ± 0.062 | 0.524 ± -0.101 | 0.648 ± 0.027 | 0.423 ± 0.032 | 0.3431 | 0.0028 | 0.0030 | 0.1871 |
| A4ul_H to A23c_L | 0.423 ± 0.060 | 0.450 ± -0.098 | 0.593 ± 0.029 | 0.352 ± 0.033 | 0.6939 | 0.0031 | 0.0189 | 0.1977 |
| A4t_L to A4ll_H | 0.610 ± 0.071 | 0.627 ± -0.107 | 0.729 ± 0.039 | 0.519 ± 0.048 | 0.8451 | 0.0064 | 0.1386 | 0.0858 |
| A4t_L to A2_H | 0.509 ± 0.080 | 0.549 ± -0.044 | 0.713 ± 0.036 | 0.505 ± 0.060 | 0.6972 | 0.0048 | 0.0253 | 0.4221 |
| A4t_L to A1_2_3tru_H | 0.577 ± 0.076 | 0.569 ± -0.080 | 0.714 ± 0.027 | 0.489 ± 0.061 | 0.9369 | 0.0046 | 0.1108 | 0.2376 |
| A4t_L to A24cd_L | 0.513 ± 0.062 | 0.450 ± -0.132 | 0.613 ± 0.029 | 0.318 ± 0.056 | 0.4705 | 0.0002 | 0.1435 | 0.1140 |
| A4t_L to A23c_L | 0.407 ± 0.062 | 0.408 ± -0.123 | 0.598 ± 0.030 | 0.284 ± 0.049 | 0.9921 | 0.0005 | 0.0094 | 0.1633 |
| A4t_H to A23c_L | 0.448 ± 0.048 | 0.481 ± -0.145 | 0.575 ± 0.040 | 0.337 ± 0.049 | 0.6341 | 0.0085 | 0.1015 | 0.0892 |
| A4tl_L to mPMtha_L | 0.416 ± 0.055 | 0.247 ± 0.179 | 0.359 ± 0.044 | 0.427 ± 0.050 | 0.0355 | 0.3227 | 0.4285 | 0.0055 |
| A4ll_H to A2_L | 0.469 ± 0.048 | 0.543 ± -0.042 | 0.595 ± 0.042 | 0.501 ± 0.052 | 0.3160 | 0.2381 | 0.0013 | 0.6120 |
| A4ll_H to A1_2_3tru_L | 0.714 ± 0.034 | 0.649 ± 0.009 | 0.823 ± 0.022 | 0.658 ± 0.048 | 0.2930 | 0.0011 | 0.0378 | 0.8766 |
| The *p* value with label ‘*’ meant the result was significant after Bonferroni correction (p < 0.05/$C_{30}^{2}$). L, lesional hemisphere; H, healthy hemisphere. Recovered group meant that motor function of SMA syndrome patients recovered within postoperative 7 days, and the unrecovered group meant did not recover. | | | | | | | | |

The *p* value with label ‘*’ meant the result was significant after Bonferroni correction (p < 0.05/$C_{30}^{2}$). L, lesional hemisphere; H, healthy hemisphere. Recovered group meant that motor function of SMA syndrome patients recovered within postoperative 7 days, and the unrecovered group meant did not recover.

| **Table S3 (continued). Functional connectivity comparison between recovered and unrecovered groups** | | | | | | | | | |
| --- | --- | --- | --- | --- | --- | --- | --- | --- | --- |
| Edge | Preoperative | | Postoperative | | *p* value  (Two sample t test) | | *p* value  (Paired t test) | | |
|  | Recovered | Unrecovered | Recovered | Unrecovered | Preoperative | Postoperative | | Recovered | Unrecovered |
|  |  |  |  |  | Recovered *vs.* Unrecovered | Recovered *vs.* Unrecovered | | Preoperative *vs.* Postoperative | Preoperative *vs.* Postoperative |
| A1_2_3ulhf_H to mPMtha_H | 0.360 ± 0.046 | 0.273 ± 0.167 | 0.417 ± 0.048 | 0.440 ± 0.050 | 0.2257 | 0.7627 | | 0.2515 | 0.0096 |
| A1_2_3tonIa_L to A2_L | 0.460 ± 0.049 | 0.672 ± -0.011 | 0.590 ± 0.039 | 0.661 ± 0.033 | 0.0011 | 0.2575 | | 0.0172 | 0.8170 |
| A1_2_3tonIa_L to A2_H | 0.518 ± 0.056 | 0.690 ± -0.054 | 0.617 ± 0.040 | 0.636 ± 0.029 | 0.0099 | 0.7854 | | 0.1097 | 0.3021 |
| A1_2_3tonIa_H to A24cd_L | 0.526 ± 0.054 | 0.576 ± -0.133 | 0.659 ± 0.023 | 0.443 ± 0.034 | 0.4383 | 0.0059 | | 0.0256 | 0.1030 |
| A1_2_3tru_L to A24cd_L | 0.499 ± 0.056 | 0.533 ± -0.102 | 0.667 ± 0.027 | 0.431 ± 0.038 | 0.6269 | 0.0045 | | 0.0086 | 0.2252 |
| A1_2_3tru_L to A23c_L | 0.490 ± 0.051 | 0.453 ± -0.087 | 0.665 ± 0.027 | 0.366 ± 0.040 | 0.5793 | 0.0003 | | 0.0066 | 0.2543 |
| A1_2_3tru_H to A24cd_L | 0.517 ± 0.058 | 0.593 ± -0.152 | 0.679 ± 0.029 | 0.441 ± 0.031 | 0.2581 | 0.0030 | | 0.0143 | 0.0383 |
| A1_2_3tru_H to A23c_L | 0.461 ± 0.052 | 0.503 ± -0.146 | 0.599 ± 0.038 | 0.357 ± 0.035 | 0.5093 | 0.0072 | | 0.0682 | 0.0653 |
| A24cd_L to A23c_L | 0.570 ± 0.048 | 0.665 ± -0.111 | 0.743 ± 0.023 | 0.554 ± 0.031 | 0.1041 | 0.0040 | | 0.0032 | 0.0642 |

| **Table S4. Characteristics of the dynamic FC matrices.** | | | | | | | | |
| --- | --- | --- | --- | --- | --- | --- | --- | --- |
| Items | Preoperative | | Postoperative | | *p* value  (Two-sample *t* test) | | *p* value  (Paired t test) | |
|  | Recovered | Unrecovered | Recovered | Unrecovered | Preoperative | Postoperative | Recovered | Unrecovered |
|  |  |  |  |  | Recovered *vs.* Unrecovered | Recovered *vs.* Unrecovered | Preoperative *vs.* Postoperative | Preoperative *vs.* Postoperative |
| Number of state 1 | 38.1 ± 10.6 | 63.3 ± 13.0 | 78.4 ± 14.7 | 74.5 ± 12.9 | 0.0383 | 0.8209 | 0.0276 | 0.5558 |
| Mean dwell time |  |  |  |  |  |  |  |  |
| State1 | 22.2 ± 8.3 | 41.7 ± 12.7 | 54.2 ± 14.6 | 57.8 ± 14.1 | 0.0740 | 0.4151 | 0.1868 | 0.8646 |
| State2 | 78.9 ± 13.0 | 35.7 ± 11.4 | 55.7 ± 13.4 | 40.8 ± 10.9 | 0.0210 | 0.4093 | 0.1208 | 0.6722 |
| Transition rates | 2.5 ± 0.6 | 3.1 ± 0.9 | 3.3 ± 0.9 | 2.8 ± 0.8 | 0.4263 | 0.8473 | 0.6018 | 0.6888 |

* The state 1 and state 2 were the results of k-means clustering analysis in dynamic FC matrices in all patients. The unit of dwell time was the number of slide window related to dynamic FC matrices. Recovered group meant that motor function of SMA syndrome patients recovered within postoperative 7 days, and the unrecovered group meant did not recover.

| **Table S5. Global properties between recovered and unrecovered groups** | | | | | | | | |
| --- | --- | --- | --- | --- | --- | --- | --- | --- |
| Global properties | Preoperative | | Postoperative | | *p* value  (Two-sample *t* test) | | *p* value  (Paired t test) | |
|  | Recovered | Unrecovered | Recovered | Unrecovered | Preoperative | Postoperative | Recovered | Unrecovered |
|  |  |  |  |  | Recovered *vs.* Unrecovered | Recovered *vs.* Unrecovered | Preoperative *vs.* Postoperative | Preoperative *vs.* Postoperative |
| Global efficiency | 0.325 ± 0.013 | 0.323 ± 0.012 | 0.360 ± 0.009 | 0.306 ± 0.016 | 0.9232 | 0.0073 | 0.0004 | 0.2659 |
| Local efficiency | 0.476 ± 0.029 | 0.456 ± 0.021 | 0.517 ± 0.018 | 0.434 ± 0.031 | 0.6063 | 0.0301 | 0.0357 | 0.3962 |
| Clustering coefficient | 0.303 ± 0.009 | 0.302 ± 0.010 | 0.321 ± 0.010 | 0.295 ± 0.016 | 0.9165 | 0.1994 | 0.1267 | 0.7072 |
| Shortest path length | 3.227 ± 0.139 | 3.252 ± 0.127 | 2.875 ± 0.079 | 3.484 ± 0.208 | 0.9005 | 0.0123 | 0.0011 | 0.2616 |
| Transitivity | 0.506 ± 0.034 | 0.495 ± 0.031 | 0.540 ± 0.029 | 0.439 ± 0.038 | 0.8198 | 0.0460 | 0.3517 | 0.2047 |
| Vulnerability | 0.134 ± 0.011 | 0.139 ± 0.010 | 0.129 ± 0.009 | 0.123 ± 0.012 | 0.7751 | 0.6660 | 0.8028 | 0.2399 |
| Fault tolerance | 1.027 ± 0.097 | 1.020 ± 0.062 | 0.845 ± 0.036 | 1.176 ± 0.121 | 0.9536 | 0.0161 | 0.0649 | 0.1942 |
| Recovered group meant that motor function of SMA syndrome patients recovered within postoperative 7 days, and the unrecovered group meant did not recover. | | | | | | | | |

| **Table S6. Nodal Efficiency between recovered and unrecovered groups** | | | | | | |  |  |
| --- | --- | --- | --- | --- | --- | --- | --- | --- |
| Nodal | Preoperative | | Postoperative | | *p* value  (Two-sample t test) | | *p* value  (Paired t test) | |
|  | Recovered | Unrecovered | Recovered | Unrecovered | Preoperative | Postoperative | Recovered | Unrecovered |
|  |  |  |  |  | Recovered *vs.* Unrecovered | Recovered *vs.* Unrecovered | Preoperative *vs.* Postoperative | Preoperative *vs.* Postoperative |
| A6dl_H | 0.145 ± 0.037 | 0.247 ± 0.041 | 0.203 ± 0.033 | 0.213 ± 0.038 | 0.0814 | 0.8501 | 0.1015 | 0.5517 |
| A6m_H | 0.173 ± 0.043 | 0.343 ± 0.036 | 0.297 ± 0.050 | 0.200 ± 0.041 | 0.0055 | 0.1543 | 0.0731 | 0.0213 |
| A6vl_H | 0.191 ± 0.031 | 0.217 ± 0.036 | 0.220 ± 0.031 | 0.165 ± 0.032 | 0.6070 | 0.2347 | 0.5654 | 0.3121 |
| A6cdl_H | 0.359 ± 0.027 | 0.293 ± 0.035 | 0.324 ± 0.045 | 0.285 ± 0.036 | 0.1601 | 0.5072 | 0.4331 | 0.8604 |
| A4ul_L | 0.472 ± 0.027 | 0.413 ± 0.032 | 0.535 ± 0.020 | 0.387 ± 0.028 | 0.1851 | 0.0002* | 0.0006* | 0.4949 |
| A4ul_H | 0.368 ± 0.028 | 0.351 ± 0.033 | 0.410 ± 0.024 | 0.384 ± 0.042 | 0.7198 | 0.5971 | 0.1712 | 0.4733 |
| A4t_L | 0.311 ± 0.044 | 0.346 ± 0.038 | 0.371 ± 0.041 | 0.322 ± 0.037 | 0.5586 | 0.3970 | 0.3128 | 0.7113 |
| A4t_H | 0.402 ± 0.039 | 0.379 ± 0.041 | 0.387 ± 0.047 | 0.320 ± 0.038 | 0.6950 | 0.2936 | 0.8072 | 0.3283 |
| A4tl_L | 0.334 ± 0.027 | 0.275 ± 0.022 | 0.285 ± 0.027 | 0.321 ± 0.036 | 0.1092 | 0.4436 | 0.1816 | 0.2908 |
| A4tl_H | 0.277 ± 0.037 | 0.252 ± 0.028 | 0.302 ± 0.029 | 0.292 ± 0.030 | 0.5971 | 0.8026 | 0.6148 | 0.3069 |
| A6cvl_L | 0.303 ± 0.030 | 0.248 ± 0.030 | 0.252 ± 0.043 | 0.230 ± 0.025 | 0.2170 | 0.6771 | 0.2215 | 0.6587 |
| A6cvl_H | 0.281 ± 0.040 | 0.243 ± 0.030 | 0.259 ± 0.033 | 0.290 ± 0.028 | 0.4674 | 0.4993 | 0.6360 | 0.3776 |
| A1_2_3ll_H | 0.301 ± 0.027 | 0.324 ± 0.041 | 0.378 ± 0.031 | 0.286 ± 0.037 | 0.6559 | 0.0706 | 0.0289 | 0.5047 |
| A4ll_H | 0.363 ± 0.033 | 0.367 ± 0.029 | 0.450 ± 0.038 | 0.246 ± 0.034 | 0.9257 | 0.0005* | 0.0488 | 0.0240 |
| A1_2_3ulhf_L | 0.407 ± 0.031 | 0.396 ± 0.030 | 0.462 ± 0.019 | 0.400 ± 0.023 | 0.8066 | 0.0505 | 0.0673 | 0.9062 |
| A1_2_3ulhf_H | 0.413 ± 0.033 | 0.399 ± 0.032 | 0.486 ± 0.015 | 0.376 ± 0.021 | 0.7766 | 0.0003* | 0.0329 | 0.5465 |
| A1_2_3tonIa_L | 0.353 ± 0.038 | 0.379 ± 0.020 | 0.367 ± 0.027 | 0.382 ± 0.023 | 0.5632 | 0.6773 | 0.7380 | 0.9038 |
| A1_2_3tonIa_H | 0.403 ± 0.035 | 0.342 ± 0.035 | 0.422 ± 0.026 | 0.367 ± 0.040 | 0.2476 | 0.2711 | 0.5772 | 0.6240 |
| A2_L | 0.309 ± 0.029 | 0.379 ± 0.025 | 0.331 ± 0.021 | 0.301 ± 0.028 | 0.0847 | 0.4270 | 0.5884 | 0.0001* |
| A2_H | 0.379 ± 0.024 | 0.388 ± 0.022 | 0.418 ± 0.023 | 0.396 ± 0.030 | 0.7836 | 0.5566 | 0.1830 | 0.8264 |
| A1_2_3tru_L | 0.468 ± 0.029 | 0.443 ± 0.021 | 0.519 ± 0.017 | 0.401 ± 0.028 | 0.5020 | 0.0016* | 0.0789 | 0.1574 |
| A1_2_3tru_H | 0.426 ± 0.027 | 0.390 ± 0.036 | 0.483 ± 0.029 | 0.349 ± 0.036 | 0.4431 | 0.0080 | 0.0598 | 0.3628 |
| A24cd_L | 0.316 ± 0.037 | 0.394 ± 0.020 | 0.397 ± 0.040 | 0.353 ± 0.040 | 0.0839 | 0.4495 | 0.1641 | 0.3224 |
| A24cd_H | 0.360 ± 0.038 | 0.368 ± 0.020 | 0.412 ± 0.037 | 0.276 ± 0.043 | 0.8529 | 0.0258 | 0.2685 | 0.0761 |
| A23c_L | 0.281 ± 0.041 | 0.271 ± 0.024 | 0.328 ± 0.030 | 0.305 ± 0.023 | 0.8444 | 0.5658 | 0.2523 | 0.3337 |
| A23c_H | 0.281 ± 0.026 | 0.283 ± 0.033 | 0.343 ± 0.037 | 0.278 ± 0.034 | 0.9640 | 0.2139 | 0.1418 | 0.9293 |
| mPMtha_L | 0.258 ± 0.031 | 0.215 ± 0.028 | 0.272 ± 0.032 | 0.204 ± 0.027 | 0.3312 | 0.1210 | 0.7060 | 0.7950 |
| mPMtha_H | 0.267 ± 0.033 | 0.231 ± 0.030 | 0.291 ± 0.027 | 0.281 ± 0.028 | 0.4409 | 0.8079 | 0.5613 | 0.2709 |
| Stha_L | 0.327 ± 0.028 | 0.271 ± 0.026 | 0.348 ± 0.027 | 0.307 ± 0.024 | 0.1538 | 0.2852 | 0.5883 | 0.3854 |
| Stha_H | 0.226 ± 0.027 | 0.250 ± 0.023 | 0.240 ± 0.034 | 0.271 ± 0.031 | 0.5082 | 0.5127 | 0.7129 | 0.5810 |
| The *p* value with label ‘*’ meant the result was significant after Bonferroni correction (p < 0.05/30). L, lesional hemisphere; H, healthy hemisphere. Recovered group meant that motor function of SMA syndrome patients recovered within postoperative 7 days, and the unrecovered group meant did not recover. | | | | | | | | |

| **Table S7. Degree Centrality between recovered and unrecovered groups** | | | | | | |  |  |
| --- | --- | --- | --- | --- | --- | --- | --- | --- |
| Nodal | Preoperative | | Postoperative | | *p* value  (Two-sample t test) | | *p* value  (Paired t test) | |
|  | Recovered | Unrecovered | Recovered | Unrecovered | Preoperative | Postoperative | Recovered | Unrecovered |
|  |  |  |  |  | Recovered *vs.* Unrecovered | Recovered *vs.* Unrecovered | Preoperative *vs.* Postoperative | Preoperative *vs.* Postoperative |
| A6dl_H | 1.760 ± 0.570 | 3.557 ± 0.770 | 1.938 ± 0.464 | 2.386 ± 0.674 | 0.0782 | 0.5993 | 0.6939 | 0.2525 |
| A6m_H | 2.390 ± 0.926 | 5.740 ± 1.035 | 5.225 ± 1.143 | 2.538 ± 0.751 | 0.0257 | 0.0658 | 0.0740 | 0.0142 |
| A6vl_H | 1.791 ± 0.419 | 2.696 ± 0.698 | 1.875 ± 0.486 | 1.608 ± 0.419 | 0.2888 | 0.6893 | 0.9118 | 0.1797 |
| A6cdl_H | 5.456 ± 0.799 | 4.737 ± 0.850 | 5.230 ± 0.989 | 4.308 ± 0.848 | 0.5540 | 0.4971 | 0.8400 | 0.6844 |
| A4ul_L | 10.178 ± 0.815 | 8.108 ± 1.022 | 11.771 ± 0.730 | 7.305 ± 0.827 | 0.1342 | 0.0004* | 0.0087 | 0.5237 |
| A4ul_H | 5.892 ± 0.896 | 5.812 ± 0.944 | 6.817 ± 0.835 | 7.663 ± 1.194 | 0.9531 | 0.5771 | 0.4166 | 0.1701 |
| A4t_L | 5.209 ± 1.008 | 6.204 ± 0.985 | 6.231 ± 0.910 | 5.391 ± 1.120 | 0.4986 | 0.5759 | 0.4525 | 0.6595 |
| A4t_H | 8.077 ± 1.033 | 7.415 ± 1.035 | 6.704 ± 1.227 | 5.507 ± 1.000 | 0.6635 | 0.4688 | 0.4052 | 0.2339 |
| A4tl_L | 4.750 ± 0.756 | 3.227 ± 0.581 | 2.982 ± 0.498 | 5.495 ± 0.861 | 0.1311 | 0.0199 | 0.1012 | 0.0581 |
| A4tl_H | 3.698 ± 1.053 | 3.068 ± 0.637 | 3.946 ± 0.832 | 3.843 ± 0.817 | 0.6233 | 0.9320 | 0.8692 | 0.4554 |
| A6cvl_L | 4.492 ± 0.840 | 2.889 ± 0.677 | 3.476 ± 1.008 | 2.559 ± 0.602 | 0.1592 | 0.4539 | 0.3304 | 0.7151 |
| A6cvl_H | 3.989 ± 1.099 | 2.882 ± 0.600 | 2.917 ± 0.646 | 3.940 ± 0.604 | 0.3973 | 0.2700 | 0.4027 | 0.2793 |
| A1_2_3ll_H | 4.471 ± 0.797 | 5.363 ± 1.052 | 6.012 ± 0.836 | 4.662 ± 0.905 | 0.5167 | 0.2959 | 0.1431 | 0.5901 |
| A4ll_H | 6.137 ± 0.847 | 6.093 ± 0.795 | 9.024 ± 0.994 | 3.357 ± 0.706 | 0.9710 | 0.0001* | 0.0236 | 0.0235 |
| A1_2_3ulhf_L | 7.677 ± 1.071 | 7.275 ± 0.933 | 8.712 ± 0.750 | 7.725 ± 0.719 | 0.7851 | 0.3632 | 0.3930 | 0.7043 |
| A1_2_3ulhf_H | 7.685 ± 1.203 | 7.651 ± 1.002 | 9.763 ± 0.681 | 6.303 ± 0.725 | 0.9834 | 0.0020 | 0.1628 | 0.3476 |
| A1_2_3tonIa_L | 5.935 ± 1.111 | 6.612 ± 0.675 | 5.538 ± 0.894 | 7.256 ± 0.771 | 0.6169 | 0.1679 | 0.7633 | 0.5436 |
| A1_2_3tonIa_H | 7.465 ± 1.186 | 5.831 ± 1.071 | 7.268 ± 1.017 | 6.944 ± 1.245 | 0.3287 | 0.8459 | 0.8823 | 0.4816 |
| A2_L | 4.443 ± 0.750 | 6.505 ± 0.895 | 4.175 ± 0.608 | 4.357 ± 0.657 | 0.0963 | 0.8444 | 0.7978 | 0.0020 |
| A2_H | 6.458 ± 0.835 | 6.897 ± 0.592 | 7.057 ± 0.896 | 7.459 ± 0.969 | 0.6803 | 0.7696 | 0.6249 | 0.6332 |
| A1_2_3tru_L | 9.998 ± 0.957 | 8.930 ± 0.726 | 11.233 ± 0.669 | 7.488 ± 0.931 | 0.3947 | 0.0034 | 0.2595 | 0.1906 |
| A1_2_3tru_H | 8.321 ± 0.884 | 7.588 ± 1.014 | 9.638 ± 1.027 | 6.089 ± 0.949 | 0.6008 | 0.0194 | 0.2928 | 0.2516 |
| A24cd_L | 5.271 ± 0.973 | 7.013 ± 0.631 | 7.263 ± 1.025 | 6.974 ± 1.047 | 0.1547 | 0.8499 | 0.1722 | 0.9689 |
| A24cd_H | 6.490 ± 1.129 | 5.877 ± 0.726 | 7.741 ± 0.924 | 4.451 ± 0.982 | 0.6605 | 0.0242 | 0.3322 | 0.3094 |
| A23c_L | 4.636 ± 1.000 | 3.071 ± 0.486 | 4.018 ± 0.769 | 3.996 ± 0.613 | 0.1816 | 0.9832 | 0.5675 | 0.3073 |
| A23c_H | 4.031 ± 0.721 | 3.828 ± 0.734 | 5.242 ± 0.871 | 3.990 ± 0.713 | 0.8495 | 0.2884 | 0.2300 | 0.8884 |
| mPMtha_L | 3.373 ± 0.618 | 2.692 ± 0.540 | 3.330 ± 0.684 | 1.824 ± 0.331 | 0.4266 | 0.0634 | 0.9600 | 0.2773 |
| mPMtha_H | 3.586 ± 0.712 | 3.092 ± 0.580 | 3.502 ± 0.635 | 3.861 ± 0.639 | 0.6055 | 0.7018 | 0.9260 | 0.4166 |
| Stha_L | 4.805 ± 0.626 | 3.716 ± 0.640 | 5.265 ± 0.756 | 4.318 ± 0.575 | 0.2469 | 0.3405 | 0.6387 | 0.5533 |
| Stha_H | 2.484 ± 0.547 | 2.904 ± 0.535 | 2.955 ± 0.711 | 3.697 ± 0.816 | 0.5981 | 0.5104 | 0.5712 | 0.3495 |
| The *p* value with label ‘*’ meant the result was significant after Bonferroni correction (p < 0.05/30). L, lesional hemisphere; H, healthy hemisphere. Recovered group meant that motor function of SMA syndrome patients recovered within postoperative 7 days, and the unrecovered group meant did not recover. | | | | | | | | |

| **Table S8. Nodal local efficiency between recovered and unrecovered groups** | | | | | | |  |  |
| --- | --- | --- | --- | --- | --- | --- | --- | --- |
| Nodal | Preoperative | | Postoperative | | *p* value  (Two-sample t test) | | *p* value  (Paired t test) | |
|  | Recovered | Unrecovered | Recovered | Unrecovered | Preoperative | Postoperative | Recovered | Unrecovered |
|  |  |  |  |  | Recovered *vs.* Unrecovered | Recovered *vs.* Unrecovered | Preoperative *vs.* Postoperative | Preoperative *vs.* Postoperative |
| A6dl_H | 0.208 ± 0.060 | 0.377 ± 0.069 | 0.318 ± 0.066 | 0.349 ± 0.083 | 0.0841 | 0.7723 | 0.1500 | 0.7663 |
| A6m_H | 0.208 ± 0.059 | 0.486 ± 0.055 | 0.426 ± 0.067 | 0.294 ± 0.073 | 0.0021 | 0.2045 | 0.0344 | 0.0840 |
| A6vl_H | 0.258 ± 0.075 | 0.258 ± 0.056 | 0.324 ± 0.078 | 0.229 ± 0.046 | 0.9999 | 0.3176 | 0.5539 | 0.7171 |
| A6cdl_H | 0.493 ± 0.052 | 0.423 ± 0.065 | 0.515 ± 0.078 | 0.436 ± 0.060 | 0.4221 | 0.4403 | 0.7170 | 0.8536 |
| A4ul_L | 0.585 ± 0.044 | 0.516 ± 0.047 | 0.626 ± 0.025 | 0.526 ± 0.054 | 0.2991 | 0.1148 | 0.3416 | 0.8602 |
| A4ul_H | 0.574 ± 0.065 | 0.464 ± 0.061 | 0.609 ± 0.056 | 0.473 ± 0.050 | 0.2426 | 0.0895 | 0.5390 | 0.8858 |
| A4t_L | 0.475 ± 0.069 | 0.472 ± 0.061 | 0.600 ± 0.068 | 0.497 ± 0.063 | 0.9728 | 0.2863 | 0.1522 | 0.8099 |
| A4t_H | 0.506 ± 0.059 | 0.541 ± 0.052 | 0.599 ± 0.073 | 0.438 ± 0.067 | 0.6718 | 0.1268 | 0.2348 | 0.2253 |
| A4tl_L | 0.593 ± 0.060 | 0.429 ± 0.046 | 0.448 ± 0.072 | 0.463 ± 0.061 | 0.0438 | 0.8800 | 0.0933 | 0.6105 |
| A4tl_H | 0.456 ± 0.064 | 0.342 ± 0.076 | 0.508 ± 0.049 | 0.418 ± 0.050 | 0.2723 | 0.2246 | 0.4458 | 0.4105 |
| A6cvl_L | 0.507 ± 0.069 | 0.360 ± 0.057 | 0.301 ± 0.070 | 0.348 ± 0.065 | 0.1219 | 0.6368 | 0.0481 | 0.8878 |
| A6cvl_H | 0.455 ± 0.074 | 0.342 ± 0.058 | 0.350 ± 0.073 | 0.478 ± 0.064 | 0.2536 | 0.2104 | 0.2777 | 0.1623 |
| A1_2_3ll_H | 0.505 ± 0.055 | 0.446 ± 0.056 | 0.649 ± 0.069 | 0.344 ± 0.050 | 0.4656 | 0.0015* | 0.0254 | 0.2726 |
| A4ll_H | 0.593 ± 0.061 | 0.607 ± 0.052 | 0.558 ± 0.051 | 0.362 ± 0.070 | 0.8652 | 0.0366 | 0.6389 | 0.0147 |
| A1_2_3ulhf_L | 0.548 ± 0.048 | 0.556 ± 0.045 | 0.616 ± 0.033 | 0.529 ± 0.044 | 0.9042 | 0.1338 | 0.1178 | 0.6385 |
| A1_2_3ulhf_H | 0.547 ± 0.049 | 0.566 ± 0.047 | 0.622 ± 0.030 | 0.554 ± 0.048 | 0.7785 | 0.2506 | 0.1401 | 0.8484 |
| A1_2_3tonIa_L | 0.521 ± 0.057 | 0.526 ± 0.032 | 0.541 ± 0.048 | 0.508 ± 0.048 | 0.9385 | 0.6350 | 0.7209 | 0.7174 |
| A1_2_3tonIa_H | 0.580 ± 0.040 | 0.454 ± 0.057 | 0.564 ± 0.053 | 0.458 ± 0.058 | 0.0873 | 0.1974 | 0.7453 | 0.9661 |
| A2_L | 0.551 ± 0.060 | 0.542 ± 0.047 | 0.512 ± 0.058 | 0.461 ± 0.059 | 0.9075 | 0.5533 | 0.6793 | 0.1073 |
| A2_H | 0.559 ± 0.053 | 0.593 ± 0.047 | 0.585 ± 0.041 | 0.548 ± 0.048 | 0.6435 | 0.5707 | 0.7125 | 0.3844 |
| A1_2_3tru_L | 0.592 ± 0.042 | 0.560 ± 0.040 | 0.643 ± 0.030 | 0.534 ± 0.044 | 0.5969 | 0.0561 | 0.2013 | 0.6152 |
| A1_2_3tru_H | 0.574 ± 0.051 | 0.522 ± 0.044 | 0.653 ± 0.041 | 0.513 ± 0.061 | 0.4632 | 0.0729 | 0.1743 | 0.8938 |
| A24cd_L | 0.461 ± 0.060 | 0.460 ± 0.033 | 0.533 ± 0.057 | 0.407 ± 0.053 | 0.9902 | 0.1287 | 0.3407 | 0.2531 |
| A24cd_H | 0.489 ± 0.056 | 0.497 ± 0.032 | 0.538 ± 0.050 | 0.348 ± 0.053 | 0.9017 | 0.0173 | 0.4916 | 0.0475 |
| A23c_L | 0.434 ± 0.069 | 0.427 ± 0.052 | 0.563 ± 0.067 | 0.489 ± 0.050 | 0.9370 | 0.4000 | 0.1377 | 0.4338 |
| A23c_H | 0.450 ± 0.059 | 0.415 ± 0.055 | 0.507 ± 0.059 | 0.383 ± 0.054 | 0.6726 | 0.1409 | 0.3648 | 0.7088 |
| mPMtha_L | 0.374 ± 0.062 | 0.349 ± 0.051 | 0.477 ± 0.073 | 0.328 ± 0.070 | 0.7659 | 0.1635 | 0.1960 | 0.8350 |
| mPMtha_H | 0.363 ± 0.061 | 0.332 ± 0.047 | 0.471 ± 0.065 | 0.378 ± 0.042 | 0.7019 | 0.2548 | 0.1357 | 0.4286 |
| Stha_L | 0.452 ± 0.051 | 0.386 ± 0.043 | 0.461 ± 0.054 | 0.471 ± 0.047 | 0.3449 | 0.8908 | 0.8818 | 0.1254 |
| Stha_H | 0.360 ± 0.052 | 0.441 ± 0.047 | 0.405 ± 0.072 | 0.439 ± 0.056 | 0.2706 | 0.7140 | 0.6166 | 0.9784 |
| The *p* value with label ‘*’ meant the result was significant after Bonferroni correction (p < 0.05/30). L, lesional hemisphere; H, healthy hemisphere. Recovered group meant that motor function of SMA syndrome patients recovered within postoperative 7 days, and the unrecovered group meant did not recover. | | | | | | | | |

| **Table S9. Nodal betweenness between recovered and unrecovered groups** | | | | | | |  |  |
| --- | --- | --- | --- | --- | --- | --- | --- | --- |
| Nodal | Preoperative | | Postoperative | | *p* value  (Two-sample t test) | | *p* value  (Paired t test) | |
|  | Recovered | Unrecovered | Recovered | Unrecovered | Preoperative | Postoperative | Recovered | Unrecovered |
|  |  |  |  |  | Recovered *vs.* Unrecovered | Recovered *vs.* Unrecovered | Preoperative *vs.* Postoperative | Preoperative *vs.* Postoperative |
| A6dl_H | 5.686 ± 3.425 | 3.457 ± 1.222 | 2.869 ± 1.753 | 2.296 ± 1.572 | 0.5563 | 0.8151 | 0.3921 | 0.6036 |
| A6m_H | 3.455 ± 1.464 | 8.790 ± 2.796 | 13.090 ± 6.231 | 4.226 ± 2.215 | 0.1108 | 0.2027 | 0.1474 | 0.2796 |
| A6vl_H | 4.195 ± 1.677 | 5.819 ± 1.807 | 3.138 ± 1.619 | 4.507 ± 2.286 | 0.5272 | 0.6387 | 0.6830 | 0.6795 |
| A6cdl_H | 15.570 ± 3.840 | 8.577 ± 3.653 | 9.188 ± 3.287 | 9.778 ± 4.050 | 0.2097 | 0.9132 | 0.1916 | 0.8002 |
| A4ul_L | 22.64 ± 5.169 | 22.360 ± 5.356 | 21.876 ± 3.017 | 14.762 ± 4.037 | 0.9711 | 0.1804 | 0.9005 | 0.2093 |
| A4ul_H | 9.627 ± 3.179 | 17.249 ± 5.318 | 8.441 ± 2.669 | 16.590 ± 4.590 | 0.2415 | 0.1463 | 0.7575 | 0.9342 |
| A4t_L | 2.946 ± 1.108 | 6.138 ± 1.865 | 3.258 ± 1.619 | 13.575 ± 4.532 | 0.1630 | 0.0457 | 0.8819 | 0.2191 |
| A4t_H | 15.889 ± 6.571 | 8.287 ± 2.314 | 5.989 ± 2.588 | 12.968 ± 4.533 | 0.2977 | 0.2039 | 0.1624 | 0.4183 |
| A4tl_L | 5.186 ± 2.664 | 7.509 ± 2.719 | 2.389 ± 0.920 | 8.885 ± 3.104 | 0.5579 | 0.0604 | 0.3828 | 0.7715 |
| A4tl_H | 4.007 ± 1.145 | 4.959 ± 2.193 | 4.898 ± 1.809 | 9.149 ± 2.608 | 0.7112 | 0.2031 | 0.7069 | 0.2119 |
| A6cvl_L | 8.066 ± 3.133 | 5.172 ± 1.907 | 4.296 ± 1.772 | 1.086 ± 0.595 | 0.4497 | 0.1055 | 0.2344 | 0.0412 |
| A6cvl_H | 7.222 ± 2.424 | 5.102 ± 1.828 | 6.152 ± 3.400 | 7.466 ± 3.210 | 0.5031 | 0.7868 | 0.8146 | 0.5114 |
| A1_2_3ll_H | 7.493 ± 2.963 | 23.059 ± 7.340 | 5.692 ± 2.157 | 17.962 ± 4.431 | 0.0654 | 0.0216 | 0.4900 | 0.5902 |
| A4ll_H | 8.183 ± 2.612 | 3.464 ± 1.167 | 14.604 ± 3.346 | 7.749 ± 2.232 | 0.1193 | 0.1080 | 0.0893 | 0.1081 |
| A1_2_3ulhf_L | 19.889 ± 5.228 | 10.995 ± 3.463 | 15.138 ± 4.071 | 15.753 ± 4.959 | 0.1784 | 0.9264 | 0.4996 | 0.4523 |
| A1_2_3ulhf_H | 20.097 ± 5.593 | 15.894 ± 5.098 | 22.063 ± 5.657 | 6.133 ± 1.820 | 0.5937 | 0.0140 | 0.7709 | 0.1387 |
| A1_2_3tonIa_L | 10.952 ± 2.334 | 16.425 ± 3.814 | 10.235 ± 2.510 | 18.679 ± 4.228 | 0.2438 | 0.1055 | 0.8133 | 0.7224 |
| A1_2_3tonIa_H | 16.129 ± 6.245 | 8.335 ± 2.405 | 15.681 ± 5.574 | 10.312 ± 3.741 | 0.2669 | 0.4435 | 0.9506 | 0.6010 |
| A2_L | 7.925 ± 2.647 | 9.824 ± 2.937 | 8.253 ± 2.512 | 5.339 ± 1.781 | 0.6445 | 0.3654 | 0.9284 | 0.2361 |
| A2_H | 16.362 ± 3.907 | 14.939 ± 4.423 | 17.446 ± 4.202 | 16.041 ± 5.158 | 0.8165 | 0.8390 | 0.8647 | 0.8788 |
| A1_2_3tru_L | 18.525 ± 3.608 | 25.502 ± 5.591 | 22.477 ± 5.678 | 14.303 ± 3.142 | 0.3167 | 0.2306 | 0.5810 | 0.0926 |
| A1_2_3tru_H | 19.867 ± 5.948 | 12.962 ± 3.404 | 12.821 ± 3.918 | 10.158 ± 2.524 | 0.3356 | 0.5832 | 0.3715 | 0.5358 |
| A24cd_L | 12.419 ± 4.490 | 31.156 ± 7.909 | 19.880 ± 5.889 | 25.690 ± 5.601 | 0.0542 | 0.4930 | 0.3159 | 0.5234 |
| A24cd_H | 13.55 ± 4.671 | 14.724 ± 5.170 | 19.692 ± 4.698 | 11.032 ± 3.506 | 0.8712 | 0.1615 | 0.0683 | 0.5597 |
| A23c_L | 8.808 ± 2.896 | 6.545 ± 2.748 | 2.699 ± 1.054 | 5.509 ± 1.720 | 0.5863 | 0.1860 | 0.0637 | 0.7864 |
| A23c_H | 6.308 ± 1.860 | 8.120 ± 3.629 | 8.048 ± 3.250 | 11.448 ± 3.014 | 0.6693 | 0.4622 | 0.6417 | 0.5152 |
| mPMtha_L | 4.256 ± 1.678 | 2.867 ± 1.526 | 1.661 ± 0.721 | 3.468 ± 1.481 | 0.5566 | 0.2949 | 0.1806 | 0.7976 |
| mPMtha_H | 7.466 ± 2.996 | 9.247 ± 2.407 | 2.751 ± 1.246 | 13.394 ± 3.676 | 0.6562 | 0.0121 | 0.2033 | 0.4149 |
| Stha_L | 9.120 ± 1.667 | 11.790 ± 3.152 | 16.554 ± 4.340 | 11.192 ± 2.662 | 0.4730 | 0.3146 | 0.0884 | 0.8984 |
| Stha_H | 2.808 ± 1.218 | 1.819 ± 0.776 | 6.794 ± 3.440 | 9.090 ± 2.817 | 0.5113 | 0.6197 | 0.2530 | 0.0288 |
| The *p* value with label ‘*’ meant the result was significant after Bonferroni correction (p < 0.05/30). L, lesional hemisphere; H, healthy hemisphere. Recovered group meant that motor function of SMA syndrome patients recovered within postoperative 7 days, and the unrecovered group meant did not recover. | | | | | | | | |

| **Table S10. Nodal clustering coefficient between recovered and unrecovered groups** | | | | | | |  |  |
| --- | --- | --- | --- | --- | --- | --- | --- | --- |
| Nodal | Preoperative | | Postoperative | | *p* value  (Two-sample t test) | | *p* value  (Paired t test) | |
|  | Recovered | Unrecovered | Recovered | Unrecovered | Preoperative | Postoperative | Recovered | Unrecovered |
|  |  |  |  |  | Recovered *vs.* Unrecovered | Recovered *vs.* Unrecovered | Preoperative *vs.* Postoperative | Preoperative *vs.* Postoperative |
| A6dl_H | 0.132 ± 0.038 | 0.239 ± 0.046 | 0.222 ± 0.049 | 0.231 ± 0.050 | 0.0903 | 0.9021 | 0.1313 | 0.9037 |
| A6m_H | 0.140 ± 0.041 | 0.302 ± 0.036 | 0.259 ± 0.041 | 0.215 ± 0.050 | 0.0069 | 0.5148 | 0.0581 | 0.2555 |
| A6vl_H | 0.163 ± 0.043 | 0.161 ± 0.036 | 0.198 ± 0.045 | 0.170 ± 0.035 | 0.9715 | 0.6371 | 0.6104 | 0.8738 |
| A6cdl_H | 0.289 ± 0.025 | 0.283 ± 0.045 | 0.309 ± 0.048 | 0.298 ± 0.040 | 0.9165 | 0.8577 | 0.6499 | 0.8084 |
| A4ul_L | 0.380 ± 0.022 | 0.331 ± 0.028 | 0.395 ± 0.017 | 0.342 ± 0.035 | 0.1971 | 0.1925 | 0.4919 | 0.7787 |
| A4ul_H | 0.347 ± 0.036 | 0.291 ± 0.039 | 0.385 ± 0.036 | 0.329 ± 0.034 | 0.3161 | 0.2798 | 0.3000 | 0.4226 |
| A4t_L | 0.336 ± 0.045 | 0.325 ± 0.039 | 0.393 ± 0.045 | 0.331 ± 0.037 | 0.8583 | 0.3137 | 0.2854 | 0.9178 |
| A4t_H | 0.328 ± 0.037 | 0.382 ± 0.035 | 0.385 ± 0.049 | 0.275 ± 0.039 | 0.3136 | 0.0979 | 0.2437 | 0.0597 |
| A4tl_L | 0.368 ± 0.038 | 0.267 ± 0.033 | 0.273 ± 0.044 | 0.315 ± 0.037 | 0.0607 | 0.4773 | 0.0709 | 0.2829 |
| A4tl_H | 0.277 ± 0.037 | 0.217 ± 0.047 | 0.314 ± 0.030 | 0.315 ± 0.035 | 0.3416 | 0.9896 | 0.3278 | 0.1592 |
| A6cvl_L | 0.319 ± 0.038 | 0.238 ± 0.040 | 0.190 ± 0.042 | 0.244 ± 0.046 | 0.1650 | 0.4067 | 0.0324 | 0.9314 |
| A6cvl_H | 0.269 ± 0.040 | 0.224 ± 0.039 | 0.237 ± 0.048 | 0.319 ± 0.039 | 0.4406 | 0.2072 | 0.5845 | 0.1381 |
| A1_2_3ll_H | 0.315 ± 0.033 | 0.275 ± 0.033 | 0.383 ± 0.032 | 0.222 ± 0.033 | 0.4097 | 0.0021 | 0.0519 | 0.3783 |
| A4ll_H | 0.395 ± 0.036 | 0.423 ± 0.031 | 0.353 ± 0.034 | 0.229 ± 0.039 | 0.5729 | 0.0257 | 0.4292 | 0.0006* |
| A1_2_3ulhf_L | 0.362 ± 0.031 | 0.375 ± 0.032 | 0.384 ± 0.024 | 0.358 ± 0.024 | 0.7769 | 0.4678 | 0.5327 | 0.6475 |
| A1_2_3ulhf_H | 0.355 ± 0.022 | 0.377 ± 0.031 | 0.390 ± 0.022 | 0.408 ± 0.029 | 0.5766 | 0.6305 | 0.1648 | 0.4798 |
| A1_2_3tonIa_L | 0.339 ± 0.033 | 0.335 ± 0.019 | 0.331 ± 0.034 | 0.332 ± 0.027 | 0.9352 | 0.9757 | 0.8155 | 0.9196 |
| A1_2_3tonIa_H | 0.353 ± 0.021 | 0.298 ± 0.037 | 0.338 ± 0.033 | 0.313 ± 0.038 | 0.2164 | 0.6345 | 0.6265 | 0.8246 |
| A2_L | 0.337 ± 0.031 | 0.369 ± 0.030 | 0.317 ± 0.034 | 0.331 ± 0.041 | 0.4741 | 0.7990 | 0.7129 | 0.3619 |
| A2_H | 0.353 ± 0.029 | 0.397 ± 0.028 | 0.378 ± 0.032 | 0.389 ± 0.030 | 0.2989 | 0.8204 | 0.5479 | 0.8042 |
| A1_2_3tru_L | 0.387 ± 0.021 | 0.378 ± 0.025 | 0.392 ± 0.018 | 0.372 ± 0.028 | 0.7804 | 0.5789 | 0.8574 | 0.8803 |
| A1_2_3tru_H | 0.375 ± 0.032 | 0.351 ± 0.028 | 0.413 ± 0.028 | 0.348 ± 0.040 | 0.5885 | 0.2039 | 0.4453 | 0.9450 |
| A24cd_L | 0.304 ± 0.038 | 0.276 ± 0.019 | 0.315 ± 0.036 | 0.259 ± 0.034 | 0.5357 | 0.2812 | 0.8309 | 0.5894 |
| A24cd_H | 0.305 ± 0.030 | 0.325 ± 0.021 | 0.310 ± 0.029 | 0.229 ± 0.037 | 0.6023 | 0.1026 | 0.9226 | 0.0525 |
| A23c_L | 0.284 ± 0.042 | 0.290 ± 0.036 | 0.342 ± 0.039 | 0.341 ± 0.036 | 0.9160 | 0.9834 | 0.2946 | 0.3600 |
| A23c_H | 0.276 ± 0.036 | 0.276 ± 0.037 | 0.314 ± 0.037 | 0.269 ± 0.039 | 0.9989 | 0.4279 | 0.3444 | 0.9058 |
| mPMtha_L | 0.238 ± 0.040 | 0.247 ± 0.040 | 0.304 ± 0.045 | 0.227 ± 0.047 | 0.8713 | 0.2574 | 0.2381 | 0.7806 |
| mPMtha_H | 0.235 ± 0.038 | 0.230 ± 0.034 | 0.293 ± 0.039 | 0.250 ± 0.028 | 0.9259 | 0.3973 | 0.2239 | 0.6396 |
| Stha_L | 0.274 ± 0.030 | 0.259 ± 0.031 | 0.268 ± 0.032 | 0.301 ± 0.024 | 0.7342 | 0.4401 | 0.9008 | 0.2017 |
| Stha_H | 0.254 ± 0.037 | 0.302 ± 0.036 | 0.245 ± 0.042 | 0.293 ± 0.034 | 0.3731 | 0.3966 | 0.8757 | 0.8759 |
| The *p* value with label ‘*’ meant the result was significant after Bonferroni correction (p < 0.05/30). L, lesional hemisphere; H, healthy hemisphere. Recovered group meant that motor function of SMA syndrome patients recovered within postoperative 7 days, and the unrecovered group meant did not recover. | | | | | | | | |

| **Table S11.** **Standard mean error of nodal efficiency between recovered and unrecovered groups based on dynamic FC matrices** | | | | | | | | |
| --- | --- | --- | --- | --- | --- | --- | --- | --- |
| Nodal | Preoperative | | Postoperative | | *p* value  (Two-sample t test) | | *p* value  (Paired t test) | |
|  | Recovered  (×10^-4^) | Unrecovered  (×10^-4^) | Recovered  (×10^-4^) | Unrecovered  (×10^-4^) | Preoperative | Postoperative | Recovered | Unrecovered |
|  |  |  |  |  | Recovered *vs.* Unrecovered | Recovered *vs.* Unrecovered | Preoperative *vs.* Postoperative | Preoperative *vs.* Postoperative |
| A6dl_H | 5.760 ± 1.154 | 4.681 ± 1.322 | 7.148 ± 1.563 | 5.311 ± 0.939 | 0.5552 | 0.3357 | 0.4606 | 0.6885 |
| A6m_H | 1.663 ± 0.448 | 1.575 ± 0.257 | 1.384 ± 0.346 | 3.128 ± 0.605 | 0.8701 | 0.0209 | 0.6703 | 0.0561 |
| A6vl_H | 9.212 ± 1.304 | 5.066 ± 1.165 | 11.029 ± 1.817 | 5.770 ± 1.340 | 0.0281 | 0.0308 | 0.4415 | 0.6581 |
| A6cdl_H | 2.748 ± 0.698 | 2.039 ± 0.526 | 2.229 ± 0.589 | 3.010 ± 0.423 | 0.4373 | 0.3039 | 0.5661 | 0.2603 |
| A4ul_L | 1.827 ± 0.764 | 2.094 ± 0.445 | 2.845 ± 0.459 | 4.369 ± 0.773 | 0.7716 | 0.1101 | 0.3442 | 0.0016* |
| A4ul_H | 1.846 ± 0.628 | 1.743 ± 0.357 | 2.365 ± 0.797 | 2.345 ± 0.402 | 0.8911 | 0.9823 | 0.5949 | 0.1825 |
| A4t_L | 3.037 ± 0.866 | 2.820 ± 1.023 | 3.447 ± 0.979 | 6.781 ± 1.197 | 0.8765 | 0.0445 | 0.6878 | 0.0374 |
| A4t_H | 2.070 ± 1.244 | 0.978 ± 0.161 | 1.809 ± 0.647 | 2.753 ± 0.609 | 0.4050 | 0.3104 | 0.8626 | 0.0112 |
| A4tl_L | 2.781 ± 0.669 | 3.485 ± 0.832 | 2.918 ± 0.589 | 4.618 ± 0.910 | 0.5266 | 0.1379 | 0.8436 | 0.3801 |
| A4tl_H | 2.670 ± 0.464 | 3.342 ± 0.797 | 4.179 ± 1.337 | 3.604 ± 0.667 | 0.4851 | 0.7115 | 0.2715 | 0.8215 |
| A6cvl_L | 8.243 ± 1.475 | 3.632 ± 1.026 | 6.555 ± 1.157 | 7.502 ± 1.646 | 0.0182 | 0.6510 | 0.4029 | 0.0183 |
| A6cvl_H | 7.765 ± 1.265 | 4.760 ± 1.836 | 8.589 ± 1.749 | 9.864 ± 2.122 | 0.2005 | 0.6560 | 0.6604 | 0.0457 |
| A1_2_3ll_H | 2.932 ± 0.905 | 3.127 ± 1.072 | 6.199 ± 2.079 | 4.900 ± 0.809 | 0.8936 | 0.5759 | 0.1756 | 0.2400 |
| A4ll_H | 0.979 ± 0.166 | 0.949 ± 0.204 | 2.540 ± 1.306 | 3.537 ± 0.653 | 0.9129 | 0.5124 | 0.2762 | 0.0035 |
| A1_2_3ulhf_L | 1.625 ± 0.275 | 2.118 ± 0.514 | 1.987 ± 0.568 | 3.531 ± 0.873 | 0.4181 | 0.1599 | 0.5320 | 0.0544 |
| A1_2_3ulhf_H | 2.199 ± 0.472 | 1.655 ± 0.317 | 1.736 ± 0.463 | 3.102 ± 0.831 | 0.3600 | 0.1732 | 0.5564 | 0.0666 |
| A1_2_3tonIa_L | 4.342 ± 1.256 | 2.097 ± 0.475 | 4.644 ± 1.599 | 3.910 ± 0.893 | 0.1147 | 0.7000 | 0.8779 | 0.1010 |
| A1_2_3tonIa_H | 2.557 ± 0.397 | 2.070 ± 0.322 | 2.850 ± 0.516 | 2.703 ± 0.426 | 0.3633 | 0.8319 | 0.5575 | 0.2797 |
| A2_L | 3.663 ± 0.599 | 2.739 ± 0.861 | 10.433 ± 1.973 | 6.574 ± 1.718 | 0.3987 | 0.1621 | 0.0031 | 0.0896 |
| A2_H | 2.787 ± 0.524 | 1.795 ± 0.277 | 5.192 ± 1.690 | 3.284 ± 1.164 | 0.1143 | 0.3738 | 0.2087 | 0.2213 |
| A1_2_3tru_L | 1.473 ± 0.479 | 2.213 ± 0.456 | 2.610 ± 0.639 | 4.971 ± 1.000 | 0.2856 | 0.0624 | 0.1903 | 0.0033 |
| A1_2_3tru_H | 1.027 ± 0.198 | 1.456 ± 0.574 | 2.039 ± 0.713 | 2.854 ± 0.515 | 0.4980 | 0.3758 | 0.2154 | 0.1129 |
| A24cd_L | 3.463 ± 0.915 | 3.019 ± 0.512 | 5.410 ± 1.339 | 5.483 ± 1.241 | 0.6836 | 0.9694 | 0.1406 | 0.1035 |
| A24cd_H | 3.190 ± 0.901 | 2.542 ± 0.540 | 3.144 ± 0.552 | 5.392 ± 1.002 | 0.5542 | 0.0657 | 0.9566 | 0.0105 |
| A23c_L | 3.284 ± 0.726 | 4.768 ± 1.383 | 5.377 ± 1.100 | 5.782 ± 1.315 | 0.3635 | 0.8202 | 0.1495 | 0.6511 |
| A23c_H | 2.618 ± 0.548 | 4.402 ± 1.169 | 4.166 ± 1.086 | 7.109 ± 1.850 | 0.1894 | 0.1926 | 0.1647 | 0.2891 |
| mPMtha_L | 3.533 ± 0.493 | 7.756 ± 1.187 | 8.430 ± 1.523 | 6.495 ± 1.550 | 0.0032 | 0.3942 | 0.0043 | 0.5813 |
| mPMtha_H | 4.002 ± 0.708 | 6.762 ± 0.991 | 6.416 ± 1.140 | 6.242 ± 0.973 | 0.0353 | 0.9109 | 0.1342 | 0.7410 |
| Stha_L | 6.223 ± 1.197 | 7.696 ± 1.462 | 10.758 ± 2.283 | 7.749 ± 1.699 | 0.4551 | 0.3128 | 0.1414 | 0.9778 |
| Stha_H | 4.579 ± 1.078 | 6.211 ± 1.085 | 6.722 ± 1.008 | 5.667 ± 1.041 | 0.3083 | 0.4851 | 0.2403 | 0.7397 |
| The *p* value with label ‘*’ meant the result was significant after Bonferroni correction (p < 0.05/30). L, lesional hemisphere; H, healthy hemisphere. Recovered group meant that motor function of SMA syndrome patients recovered within postoperative 7 days, and the unrecovered group meant did not recover. | | | | | | | | |

**Table S12. The mediation effect of nodal efficiency of A4ul_L between *d*_A4ul_L_ and recovery time**

|  | Effect | SE or Boot SE | t value | *p* value | Lower 95% CI | Upper 95% CI | Percentage of effect |
| --- | --- | --- | --- | --- | --- | --- | --- |
| Correlated to nodal efficiency of A4ul_L | | | |  |  |  |  |
| Constant | 0.3587 | 0.0334 | 10.7393 | < 0.0001 | 0.2906 | 0.4267 | - |
| *d*_A4ul_L_ | 0.0062 | 0.0015 | 4.0869 | 0.0003 | 0.0031 | 0.0093 | - |
| Direct effect |  |  |  |  |  |  |  |
| Constant | 41.8629 | 8.2534 | 5.0722 | < 0.0001 | 25.0297 | 58.6962 | - |
| *d*_A4ul_L_ | -0.6356 | 0.2163 | -2.9389 | 0.0062 | -1.0767 | -0.1945 | - |
| Nodal efficiency of A4ul_L | -32.4012 | 20.3593 | -1.5915 | 0.0122 | -73.9251 | -9.1227 | - |
| Total effect |  |  |  |  |  |  |  |
| Constant | 31.2407 | 3.9422 | 7.9248 | < 0.0001 | 23.2106 | 39.2708 | - |
| *d*_A4ul_L_ | -0.8437 | 0.1797 | -4.6958 | < 0.0001 | -1.2097 | -.4777 | - |
| Summary |  |  |  |  |  |  |  |
| Total effect | -0.8437 | 0.1797 | -4.6958 | < 0.0001 | -1.2097 | -0.4777 | - |
| Direct effect | -0.6356 | 0.2163 | -2.9389 | 0.0062 | -1.0767 | -0.1945 | 75.33% |
| Indirect effect | -0.2081 | 0.1010 | - | < 0.05 | -0.4336 | -0.0281 | 24.67% |

* A4ul_L, lesioned-hemispheric upper limb region BA 4. CI, confidence interval. *d*_A4ul_L_, shortest distance from surgical region to node A4ul_L. L, lesional hemisphere.
